# Systemic Hyperalgesia in Females with Gulf War Illness, Chronic Fatigue Syndrome and Fibromyalgia

**DOI:** 10.1038/s41598-020-62771-9

**Published:** 2020-04-01

**Authors:** Amber A. Surian, James N. Baraniuk

**Affiliations:** Division of Rheumatology, Immunology and Allergy, Georgetown University, 3800 Reservoir Road NW, Washington, DC 20007-2197 USA

**Keywords:** Diagnostic markers, Neuropathic pain

## Abstract

Pain is a diagnostic criterion for Gulf War Illness (GWI), Chronic Fatigue Syndrome (CFS), and fibromyalgia (FM). The physical sign of systemic hyperalgesia (tenderness) was assessed in 920 women who were stratified by 2000 Kansas GWI, 1994 CFS, and 1990 FM criteria. Pressure was applied by dolorimetry at 18 traditional tender points and the average pressure causing pain determined. GWI women were the most tender (2.9 ± 1.6 kg, mean ± SD, n = 70), followed by CFS/FM (3.1 ± 1.4 kg, n = 196), FM (3.9 ± 1.4 kg, n = 56), and CFS (5.8 ± 2.1 kg, n = 170) compared to controls (7.2 ± 2.4 kg, significantly highest by Mann-Whitney tests p < 0.0001, n = 428). Receiver operating characteristics set pressure thresholds of 4.0 kg to define GWI and CFS/FM (specificity 0.85, sensitivities 0.80 and 0.83, respectively), 4.5 kg for FM, and 6.0 kg for CFS. Pain, fatigue, quality of life, and CFS symptoms were equivalent for GWI, CFS/FM and CFS. Dolorimetry correlated with symptoms in GWI but not CFS or FM. Therefore, women with GWI, CFS and FM have systemic hyperalgesia compared to sedentary controls. The physical sign of tenderness may complement the symptoms of the Kansas criteria as a diagnostic criterion for GWI females, and aid in the diagnosis of CFS. Molecular mechanisms of systemic hyperalgesia may provide new insights into the neuropathology and treatments of these nociceptive, interoceptive and fatiguing illnesses.

## Introduction

Pain and tenderness (systemic hyperalgesia) are common complaints in Gulf War Illness (GWI)^1–3^, Chronic Fatigue Syndrome (CFS)^4^, and fibromyalgia (FM)^5–8^. The symptoms of myalgia and arthralgia are in the diagnostic criteria for all three conditions, but it is not clear if pain severity can distinguish between them. This ambiguity is of importance because (i) pain was not included as a distinguishing feature in the most recent reconceptualization of CFS as Systemic Exertion Intolerance Disease (SEID)^9^, (ii) fatigue, unrefreshing sleep, and cognitive dysfunction were added to the 2010^6^ and 2011^7^ American College of Rheumatology criteria for FM, (iii) pain, fatigue, sleep and cognitive dysfunction are defining characteristics of GWI^2,3^. Symptoms alone are insufficient to distinguish between these diseases.

An alternative approach to distinguish between GWI, CFS and FM may be to assess the sign of systemic hyperalgesia, the perception of physical discomfort elicited by pressure stimulation^10^. Assessment of tenderness was only required for the 1990 FM criteria^5^. Dolorimetry (algometry) was used to quantify the cutaneous pressure required to induce pain^11–13^. This semi-quantitative method provides a more robust and reproducible measurement than traditional tender point counts that are influenced by psychological status^14,15^.

A confounding design in studies of hyperalgesia is to compare dolorimetry between tender (e.g. defined using 1990 FM criteria^5^) versus nontender (general population) groups. Such a study design ensures a floor effect for pressures that cause pain in FM, a ceiling effect in the control group, and demarcation of a threshold of ~4 kg for separating the 2 groups. We avoided this confound by not using tenderness to define CFS or GWI status.

Systemic hyperalgesia may be present to some extent in CFS^16^ and GWI^17^, and so tenderness was stratified by comparison to sedentary control (SC) women. Females were studied because preliminary data suggested a sexual dimorphism.

A series of clinical research studies were designed to prospectively incorporate questionnaires about pain^18^, fatigue^19^, quality of life^20^ and other variables^21^, history of GWI^2,3^, CFS^4^ and FM^5^, and physical examination for tender point counts and dolorimetry^5,13,14,16^. CFS was defined using the 1994 Center for Disease Control (CDC) “Fukuda” criteria^4,22–24^. GWI was defined by 1998 Center for Disease Control criteria for Chronic Multisymptom Illness (CMI)^2^ plus the 2000 Kansas criteria^3^. FM was defined using the 1990 American College of Rheumatology criteria of widespread pain plus tenderness to thumb pressure at ≥11 of 18 traditional tender points^5^. Subjects were excluded if they had chronic medical or psychiatric diseases that were exclusionary for CFS^22–26^. Three approaches were used to classify the women into GWI, CFS alone, FM alone, overlapping CFS plus FM (CFS/FM), and sedentary control (SC) groups. Pressure-induced pain measurements were compared to the severities of myalgia, arthralgia, fatigue, quality of life, and other variables. We proposed that the distributions of systemic hyperalgesia would stratify these clinical groups despite their symptomatic overlap. If so, neural mechanisms of systemic hyperalgesia may contribute to disease morbidity. The clinical implication was that the physical sign of tenderness may have potential as a diagnostic criterion in these conditions.

## Results

The 920 women who were qualified for the study were assessed using 3 approaches. GWI women were deployed to the 1990-1991 Persian Gulf War and met both Chronic Multisymptom Illness (CMI)^2^ and Kansas criteria^1,3^ (n = 70). Participants who did not meet GWI, 1994 CDC CFS^4^ or 1990 ACR FM^5^ criteria were considered to be healthy sedentary control females (SC, n = 428). By the nature of their recruitment, SC included some subjects with chronic idiopathic fatigue, other disorders, and those with low dolorimetry thresholds without widespread pain. The remaining 422 women met 1994 Fukuda criteria for CFS^4^ and/or 1990 FM criteria of widespread pain plus tenderness at ≥11 of 18 traditional tender points^5^. In Approach 1, subjects were stratified by 1994 CFS criteria to generate groups of CFS_1994_ (n = 366, met 1994 CFS ± 1990 FM criteria) and FM_1994_ (n = 56, met 1990 FM but not 1994 CFS criteria). Approach 2 applied 1990 FM criteria [5] to select FM_1990_ (n = 252, met 1990 FM ± 1994 CFS criteria) and CFS_1990_ (n = 170, met 1994 CFS criteria but not 1990 FM criteria). GWI (n = 70) and SC (n = 428) groups were the same for each approach. The results of Approaches 1 and 2 were discussed in Supplementary Online Material. The major finding was the large overlap group who met both 1994 CFS and 1990 FM criteria (CFS/FM). Therefore, the final main approach defined 5 groups from the combination of the 3 diagnostic criteria: (i) GWI, (ii) CFS only, (iii) FM only, (iv) CFS plus FM (CFS/FM), and (v) SC.

In Approach 1, the 1994 CDC criteria^4^ selected CFS_1994_ (n = 366), FM_1994_ (n = 56), GWI (n = 70) and SC (n = 428) groups before dolorimetry was assessed (Supplementary Table S1). The diagnoses of CFS and GWI did not require tenderness, but both groups had significant systemic hyperalgesia compared to SC (Supplementary Fig. S1, Table S2). GWI had significantly lower dolorimetry pressures, higher tender point counts and McGill Total Pain scores than the CFS_1994_ group. The CFS_1994_ and FM_1994_ groups had equivalent systemic hyperalgesia. Quality of Life and other subjective scores were equivalent in GWI and CFS_1994_ and indicated significantly more impairment than the FM_1994_ and SC_1994_ groups (Supplementary Figs. S2 to S4). Only the GWI group had correlations with explained variances R^2^ > 0.25 between dolorimetry and symptom scores (Supplementary Table S3). Age did not correlate with dolorimetry in any group in Approach 1 (Supplementary Fig. S5).

Approach 2 used the 1990 FM criteria of widespread pain and tenderness to thumb pressure^5^ to select the FM_1990_ (n = 252), CFS_1990_ (n = 170), GWI (n = 70) and SC (n = 428) groups. The dolorimetry results were shifted to the left for GWI and FM_1990_ and the threshold defined by receiver operating characteristics (ROC) remained at 4.5 kg (Supplementary Tables S4 and S5). The CFS_1990_ group had a rightward shift towards the sedentary control group compared to CFS_1994_, and an increase in dolorimetry threshold to 6 kg by ROC (Fig. S6). GWI had worse McGill Total Pain scores than CFS_1990_, FM_1990_ and SC groups (Supplementary Table S4). However, scores for CFS Severity, MDFI and SF-36 quality of life domains were equivalent between GWI, CFS_1990_, and FM_1990_ and significantly worse than the SC group (Supplementary Figs. S7 to S9). Again, only GWI had explained variances with R^2^ > 0.25 for dolorimetry and symptoms (Supplementary Table S6).

These outcomes indicated that biases were introduced when using only the 1994 CFS or 1990 FM criteria to select study participants because there was a large group of women who met both CFS and FM criteria (“CFS/FM”, n = 196).

For the main approach, 1994 CFS^4^ and 1990 FM^5^ criteria were applied to select CFS (n = 170, not 1990 FM), CFS/FM (n = 196, both 1994 CFS and 1990 FM), FM (n = 56, not 1994 CFS), GWI and SC. Average age was in the 5^th^ decade, but GWI women were older than SC (Table 1). CFS had the highest proportion of Caucasians. All 70 GWI females met CFS criteria, and 60 met FM criteria.

**Table 1:** Stratification and demographics. Subjects were divided based on 2000 Kansas GWI^2,3^, 1994 CFS^4^, and 1990 FM criteria^5^. Differences between outcomes (mean ± SD) were compared by ANOVA followed by Tukey Honest Significant Difference and nonparametric Kruskal-Wallis and Mann-Whitney tests for dolorimetry (p < 0.05).

Entry criterion	Females with dolorimetry measurements				
Exclusions	Chronic medical or psychiatric diseases				
GWI status^1–3^	Gulf War exposures in 1990 & 1991 + Kansas GWI criteria [3]				
	Yes	No			
	GWI	Not GWI			
CFS status Fukuda Criteria, 1994^4^		6 months of disabling fatigue without explanation plus ≥4 of 8 ancillary criteria [4]			
	Yes	Yes	No	Yes	No
	GWI	CFS	Not CFS	CFS	Not CFS
1990 FM status^5^ Not assessed a priori		Widespread pain + Tender points by thumb pressure [5]			
		≥11/18 tender points		<11/18 tender points	
		Yes	Yes	No	No
**Groups**	**GWI**	**CFS/FM**	**FM**	**CFS**	**SC**
N	70	196	56	170	428
Age (years)	48.2 ± 11.4*	45.5 ± 12.1	46.4 ± 14.0	45.0 ± 10.9	42.7 ± 13.5
% Caucasian	65.2%	77.7%	64.4%	85.6%	60.2%
Dolorimetry (kg)	2.9 ± 1.6*,^†^	3.1 ± 1.4*,^†^	3.9 ± 1.4*,^†^	5.8 ± 2.1*	7.2 ± 2.4
Mann-Whitney tests vs SC	p < 0.0001	p < 0.0001	p < 0.0001	p < 0.0001	Kruskal-Wallis k = 5 p < 0.0001
Mann-Whitney tests vs CFS	p < 0.0001	p < 0.0001	p < 0.0001	Kruskal-Wallis k = 4 p < 0.0001	excluded
Mann-Whitney tests vs FM	p < 0.0001	p < 0.0001	Kruskal-Wallis k = 3 p < 0.0001	excluded	excluded
Mann-Whitney tests vs CFS/FM	p = 0.071	excluded	excluded	excluded	excluded
Range	0.2 to 7.2	0.5 to 8.3	0.6 to 9.8	1.4 to 12.5	0.4 to 12.5
Median	2.6	2.9	3.6	5.5	6.9
1^st^ quartile	1.8	2.0	2.9	4.4	5.5
3^rd^ quartile	3.5	3.7	4.4	6.6	8.8
Skewness	0.936	0.911	1.542	0.748	0.125
Kurtosis	0.555	1.423	5.658	0.583	−0.580
Tender point counts (0-18)	12.7 ± 5.1*,^†^	13.3 ± 4.7*,^†^	11.4 ± 4.6*,^†^	5.0 ± 3.7*	3.3 ± 3.9
McGill Pain Total Score	21.5 ± 11.9*	15.6 ± 9.1*,^‡^	9.4 ± 7.6^‡^	12.2 ± 8.7*,^‡^	3.2 ± 6.3^‡^
	n = 57	n = 64	n = 18	n = 26	n = 75

Significantly different by ANOVA and Tukey HSD < 0.05 compared to: *SC, ^†^CFS, ^‡^GWI.

The coefficient of variability for dolorimetry was 9.3% for 57 women who had serial measurements on 3 days by different staff members. The Pearson correlation coefficient between thumb pressure tender point counts and dolorimetry pressure thresholds was −0.862 for all subjects (explained variance = 0.742). Dolorimetry distributions were not normal by one-sample Kolmogorov-Smirnov tests with Lilliefois corrections (p < 0.028) but were skewed to the right (Table 1). Kruskal-Wallis and Mann-Whitney tests were significant (p < 0.0001) for all comparisons except GWI vs. CFS/FM indicating that every subgroup had significant systemic hyperalgesia compared to SC. Tenderness was reported as median with 1^st^ and 3^rd^ quartiles.

**Figure 1:**
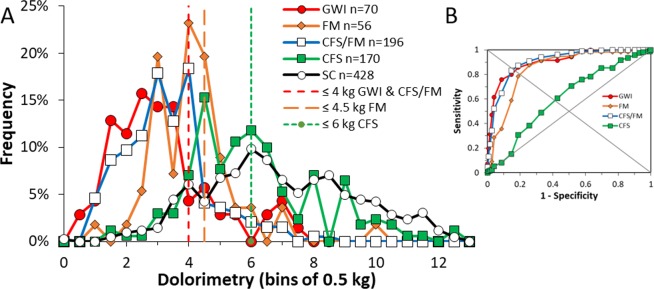
Dolorimetry frequency analysis using bins of 0.5 kg. The distributions of average pressure thresholds causing pain (**A**) were shifted to the left in GWI (red diamonds and line), CFS/FM (blue triangles and line) and FM (orange diamonds and line) compared to SC (white circles, black line) females. The distribution for CFS overlapped the SC group but was significantly different by Mann-Whitney test (p < 0.0001). Thresholds of 4.0 kg for GWI and CFS/FM (vertical red dashed line), and 4.5 kg for FM (vertical orange dashed line) were defined by ROC (**B**).

Bins of 0.5 kg were used to rank subjects for frequency analysis. SC females had a very wide and squat frequency distribution (7.2 ± 2.4 kg, mean ± SD, Table 1) that extended from 0.4 to 12.5 kg (Fig. 1). Dolorimetry pressure levels (kg) were significantly lower in GWI, CFS/FM and FM than SC (Tukey HSD < 0.05), while CFS had an intermediate level that was also significantly lower than SC (p < 0.0001 by Mann-Whitney test, Table 1). ROC defined a dolorimetry threshold of ≤4.0 kg for GWI (sensitivity = 0.800, AUC = 0.905) and CFS/FM (sensitivity = 0.832, AUC = 0.906) with specificities = 0.853 (Supplementary Table S7). Sensitivity and specificity for GWI were slightly reduced because of a bimodal distribution with about 10% of women in a second peak with pressure thresholds at 6 to 8 kg (Fig. 1). The threshold for FM was 4.5 kg (sensitivity = 0.786, specificity = 0.811, AUC = 0.848), and ≤6.0 kg for CFS (sensitivity = 0.645, specificity = 0.645, AUC = 0.672) (Fig. 1).

**Figure 2:**
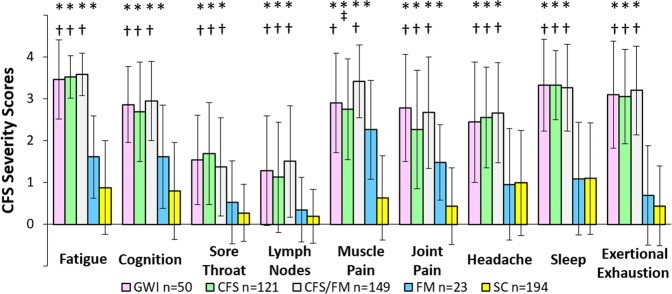
Chronic Fatigue Syndrome Symptom Severity Questionnaire [21]. GWI, CFS and CFS/FM had significantly worse symptoms over the past 6 months (mean ± SD) compared to SC (*) and FM (†). CFS/FM (‡) had significantly worse myalgia than CFS. ANOVA (p < 0.05) was followed by Tukey’s Honest Significant Difference (p < 0.010) plus FDR (p < 0.005) to correct for all data comparisons.

GWI, CFS and CFS/FM had equivalent scores for the 9 Chronic Fatigue Syndrome Symptom Severity questionnaire items (Fig. 2)^21^. Scores were significantly higher than both FM and SC. FM had intermediate scores that were greater than SC for Fatigue, Cognition and Arthralgia. Myalgia was an exception because CFS/FM scores were higher than both CFS and FM, while FM scores were elevated and equivalent to CFS.

**Figure 3:**
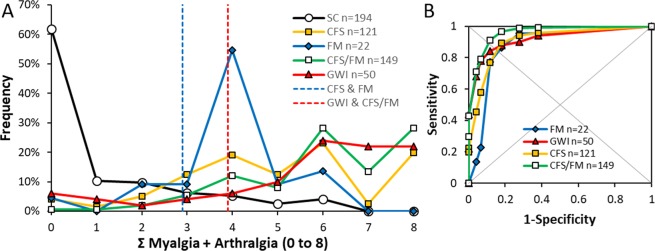
Sum of myalgia plus arthralgia scores. (**A**) The frequency distribution for SC (black line, white circles) was shifted to the left (floor effects compared to FM (blue line and diamonds), CFS (yellow line and squares), CFS/FM (green line and white squares), and GWI (red line and triangles). (**B**) Threshold scores of ≥3 for CFS and FM (vertical blue dashed line), and ≥4 for GWI and CFS/FM (vertical red dashed line) were defined by ROC.

The sum of Myalgia and Arthralgia scores was calculated as a proxy for total body musculoskeletal pain symptoms. SC had a floor effect with low scores of 0 to 2 in 82% of subjects (Fig. 3). The FM group had a mode of 4, and was distinguished from SC by a threshold of ≥3 (86.4% sensitivity, 82.0% specificity). CFS also had a threshold of ≥3 but had a broader range of scores (89.3% sensitivity, 82.0% sensitivity). The GWI and CFS/FM groups had the highest scores with a threshold of ≥4 and sensitivities of 84.0% and 91.3%, respectively, and specificities of 88.1%. Overall, a score of 4 out of 8 had sensitivity of 84.2% and specificity of 88.1% for selecting subjects with pain. These results establish that muscle and joint pain are significant components of GWI, CFS and FM.

**Figure 4:**
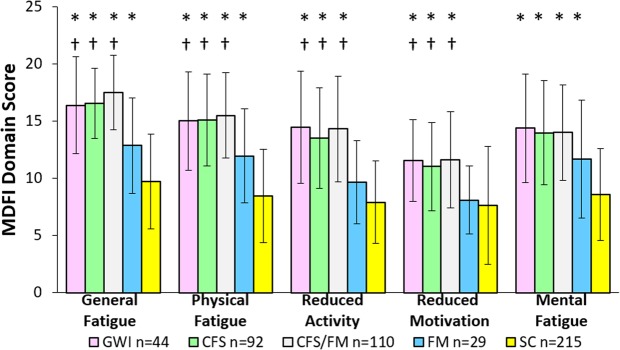
Multidimensional Fatigue Inventory Domain scores (mean ± SD) [19]. GWI (pink), CFS (green) and CFS/FM (grey) had significantly worse fatigue than SC (* yellow) and FM († blue) using ANOVA (p < 0.05) followed by Tukey’s Honest Significant Difference (p < 0.010) plus FDR (p < 0.005) to correct for all data comparisons.

Multidimensional Fatigue Inventory domain scores were equivalent for GWI, CFS and CFS/FM, and significantly higher than FM and SC (Fig. 4) for General Fatigue, Physical Fatigue, Reduced Activity and Reduced Motivation. SC had significantly lower Mental Fatigue than the other 4 groups.

**Figure 5:**
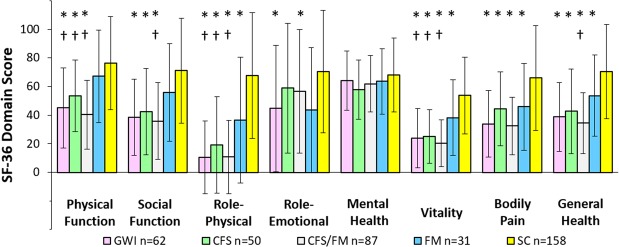
SF-36 domain scores (mean ± SD) [20]. Physical Function, Role Physical and Vitality were significantly worse for GWI, CFS and CFS/FM compared to SC (*) and FM (†) by ANOVA followed by Tukey’s Honest Significant Difference (p < 0.05) plus FDR (p < 0.005) to correct for all data comparisons.

Physical Function, Role Physical and Vitality domain scores of the SF-36 quality of life survey were equivalent for GWI, CFS and CFS/FM, and significantly worse than both SC and FM groups (Fig. 5). Bodily Pain, Social Function and General Health were also equivalent for these 3 groups and worse than SC. Mental Health was equivalent between all groups. Wide variances for the SC group reflect the inclusion of chronic idiopathic fatigue and chronic rhinosinusitis subjects in the sedentary control group.

Pain thresholds (kg) measured by dolorimetry were highly correlated with the number of tender points determined by thumb pressure, particularly in the FM and SC groups that were distinguished by the presence or absence, respectively, of tenderness to pressure (Table S8). Only the GWI group had other correlations with R > 0.5 (R^2^ > 0.25) for dolorimetry versus joint pain, muscle pain, exertional exhaustion, fatigue, Physical Functioning, Bodily Pain, Social Functioning, Reduced Activity and Physical Fatigue. Important negative findings were the absence of correlations between pain thresholds and age, sleep, cognition, and Mental Health.

Dolorimetry pain thresholds (kg) were correlated with the number of tender points determined by thumb pressure in all 4 groups (Table 2). However, only GWI had meaningful correlations (R^2^ > 0.2) with pain, physical functioning and other variables.

**Table 2:** Main approach explained variances (R^2^) from Pearson correlations between dolorimetry (kg) and domain scores for each group.

	GWI	CFS	CFS/FM	FM	SC
Tender point count	0.479	0.591	0.544	0.633	0.629
≥11/18 tender points	0.326	0.022	0.359	0.415	0.212
McGill Total Pain Score	0.320	0.122	0.173	0.149	0.112
**SF-36**					
Physical Functioning	0.354	0.011	0.167	0.001	0.000
Bodily Pain	0.328	0.005	0.093	0.000	0.005
Social Functioning	0.271	0.000	0.096	0.026	0.000
Role-Physical	0.245	0.050	0.011	0.038	0.003
Role-Emotional	0.164	0.014	0.000	0.000	0.004
General Health	0.121	0.012	0.071	0.009	0.001
Mental Health	0.098	0.095	0.001	0.001	0.001
Vitality	0.063	0.002	0.096	0.002	0.003
**CFS Symptom Severity Scores**					
Joint pain	0.457	0.001	0.039	0.002	0.031
Muscle pain	0.437	0.034	0.125	0.178	0.054
Exertional exhaustion	0.377	0.013	0.037	0.213	0.007
Fatigue	0.256	0.003	0.018	0.055	0.020
Disturbed sleep	0.162	0.000	0.044	0.149	0.009
Throat	0.145	0.000	0.008	0.003	0.033
Sore lymph nodes	0.101	0.008	0.024	0.085	0.009
Headache	0.081	0.048	0.000	0.158	0.001
Memory & concentration	0.020	0.001	0.052	0.025	0.002
**Multidimensional Fatigue Inventory**					
Reduced Activity	0.302	0.005	0.016	0.000	0.014
Physical Fatigue	0.269	0.005	0.033	0.023	0.032
Reduced Motivation	0.188	0.002	0.006	0.002	0.018
General Fatigue	0.180	0.001	0.017	0.001	0.000
Mental Fatigue	0.050	0.019	0.016	0.019	0.001
Age	0.006	0.018	0.010	0.001	0.000

The Pearson correlations between the sum of myalgia and arthralgia scores (Fig. 3) and kg by dolorimetry found a significant correlation for GWI (R^2^ = 0.515), but low explained variances for the other groups (Supplementary Fig. S10). SC showed floor effects (R^2^ = 0.055). The correlation for myalgia plus arthralgia scores with tenderness in GWI suggests the hypothesis that mechanisms of systemic hyperalgesia contribute to pain perception in GWI females.

**Figure 6:**
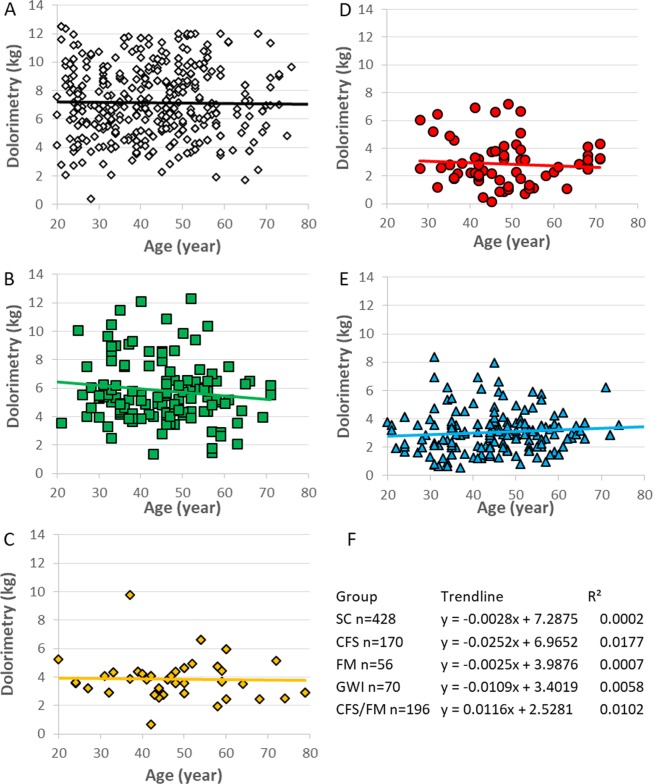
Age distributions of dolorimetry pain thresholds. The y-intercepts (kg) from linear regression lines were used to rank groups as: SC > CFS > FM > GWI > CFS/FM (**A–E**). All regression lines were horizontal with R^2^ < 0.018 indicating no significant correlations for dolorimetry with age (**F**).

Age did not correlate with dolorimetry in any of these cross-sectional groups as shown by the horizontal regression lines in Fig. 6. This suggested systemic hyperalgesia thresholds did not increase with age in these groups. Dolorimetry thresholds were highest in SC women over the entire age range. GWI and CFS/FM had the greatest tenderness.

## Discussion

Systemic hyperalgesia was found for GWI (Fig. 1)^17^, CFS defined by 1994 criteria^16^ (Fig. S1), and FM when defined using 1990 criteria (Fig. S6)^5^. Approaches 1 and 2 (Supplementary Online Materials) demonstrated the biases introduced by using only the 1994 CFS or 1990 FM criteria, respectively, as the primary selection instrument for case designation, and the large contribution by subjects who met both criteria. This confound was avoided in our main approach by stratifying subjects into CFS/FM, CFS and FM. As a result, dolorimetry thresholds in kg were ranked as SC > CFS > FM > CFS/FM > GWI (Table 1, nonparametric tests). In contrast, symptom severities and impairment were ranked GWI = CFS/FM = CFS > FM > SC (Figs. 2–5). Therefore, the two groups with the greatest tenderness, GWI and CFS/FM, also had the worst symptom scores. However, only GWI had dolorimetry pressure thresholds that correlated with symptom severities (Table 2). The CFS and FM subgroups did not have correlations between symptoms and systemic hyperalgesia. This suggests that the neural mechanisms for self-reporting perceptions of painful sensations are different from the gating mechanisms for nociceptive signal transmission in systemic hyperalgesia.

**Figure 7:**
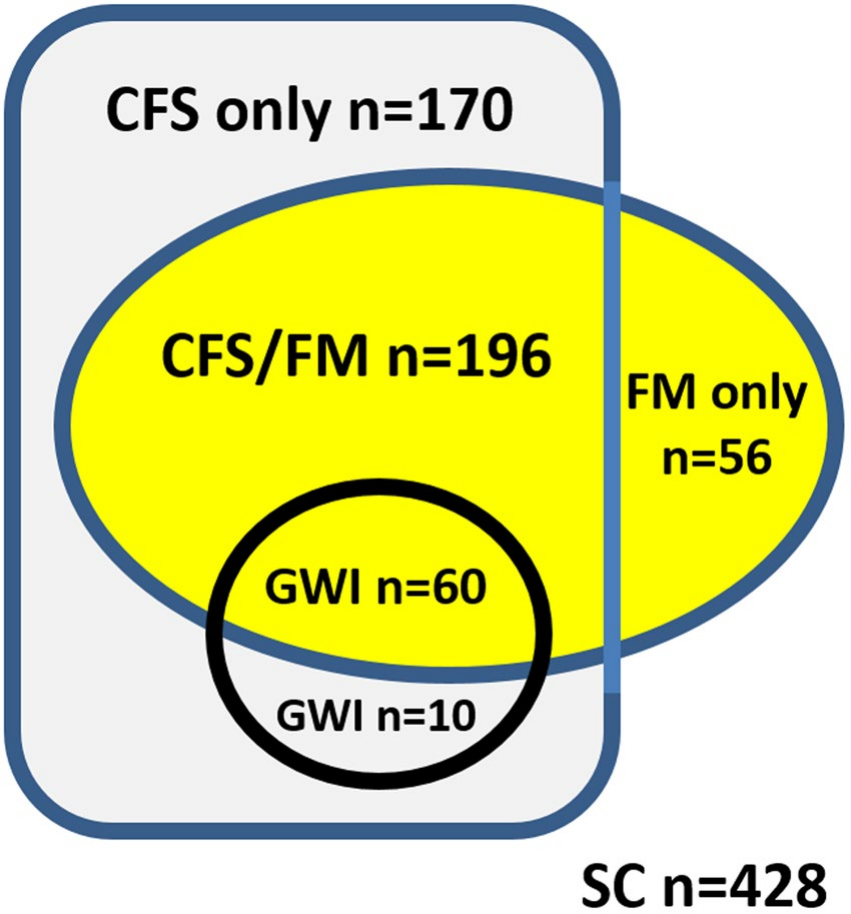
Venn diagram of main approach. GWI and CFS/FM females had the greatest tenderness and worst symptom severities. However, systemic hyperalgesia and symptoms were only correlated for GWI women.

Recognition of the sizable CFS/FM group is important for reconciling the 1994 CFS criteria with the pain and tenderness of the 1990 FM criteria, and the overlap of pain, fatigue, cognition, sleep and somatic complaints in the 2010 and 2011 FM criteria (Table 3). The CFS/FM group is relevant to the 2015 SEID criteria^9^ that did not include pain symptoms because pain was not unique to CFS. However, the CFS/FM group (54% of CFS subjects, Fig. 7) indicated systemic hyperalgesia and pain are important components of the CFS experience that should be addressed in the clinical management of CFS subjects. There have been many more clinical trials of antinociceptive drugs in FM than CFS; the overlap group may provide a rationale for using these drugs for CFS patients who also meet 1990 FM criteria^5^. Approach 2 found significant pain in CFS_1990_ CFS women^4^ who did not meet 1990 FM criteria or have systemic hyperalgesia; they may also benefit from these drugs despite the lack of clinical trials in CFS. Of interest was the subset of FM subjects who had pain without tenderness indicating that mechanisms regulating the self-report of pain and systemic hyperalgesia may be unlinked in this subgroup^27^. CFS/FM was 78% of the 1990 FM group, but this is probably an overestimate because we did not specifically recruit subjects who self-identified as FM. The poor correlations between dolorimetry and subjective measures of pain (Supplementary Table S6) demonstrate that pain and systemic hyperalgesia were not synonymous pathological processes, and may have distinct mechanisms and responses to therapies. In general, GWI, CFS/FM and CFS groups had equivalent subjective complaints that were significantly worse than the FM only group (Figs. 2–5).

**Table 3:** Overlap of diagnostic criteria.

Chronic Multi-symptom Illness (CMI) 1998	GWI “Kansas” 2000	CFS “Fukuda” 1994	FM 1990	FM 2010	SEID 2015
			Tenderness		
Musculo-skeletal	Pain	Myalgia	Widespread Pain	Widespread Pain	
		Arthralgia			
Fatigue	Fatigue, Sleep	Fatigue		Fatigue	Fatigue
		Sleep		Waking unrefreshed	Waking unrefreshed
	Post-exertional malaise	Post-exertional malaise			Post-exertional malaise
Cognition, Mood	Cognition, Mood, Neurological	Cognition		Cognition	Cognition
		Headache			
	Gastrointestinal			Somatic symptoms*	
	Respiratory	Sore throat			
	Skin	Sore lymph nodes			Orthostatic Intolerance
≥1 chronic symptom in ≥2 categories	≥3 of 6 categories	Fatigue plus ≥4 of 8	Pain + Tenderness	Severity scores	Moderate or severe >50% of time

*Replaced by headache, feeling depressed and abdominal pain in 2011^7^.

The systemic hyperalgesia in GWI women generated the hypothesis that dolorimetry pressure thresholds may be a biomarker of GWI in females exposed to the conditions of the 1990–1991 Persian Gulf War^1^. This hypothesis can be tested in Department of Veterans Affairs Medical Centers and other clinical systems by using dolorimetry as a Common Data Element in longitudinal and epidemiological studies, and by incorporating this measurement into the standard clinical physical examination. Techniques of thumb pressure for tender point counts and dolorimetry are skills that would need to be taught to generalists, nurses, physiotherapists or specialists working with GWI veterans^11–14,16,28^. Future studies may determine that there is a smaller set of tender points or other regions such as the thumb nail bed that are suitable for mass screening of systemic hyperalgesia. Other standardized methods of pressure testing may be more easily accommodated into clinics^29^. Heat^11,30^ or other modalities may be alternative stimuli for testing systemic hyperalgesia. Neural plasticity and disruption of somatosensory and interoceptive sensing and regulatory pathways in GWI, CFS, and allied disorders^31–35^ may contribute to mechanisms of migraine^17,36^, nonallergic rhinitis^16,37–39^, dyspnea^40^, pelvic pain, and other interoceptive discomfort that is referred from mucosal and visceral organs. Adaptation of research methods or creation of innovative devices that access these sensory modalities and organs may provide additional options for studying systemic hyperalgesia and allodynia in these diseases.

Questionnaires addressing the symptom severities of the Kansas^41^ and CFS^21^ criteria can be adapted for use on electronic health record dashboards for physicians during routine clinic visits, and in larger clinical research studies to determine if there are clusters of symptoms plus systemic hyperalgesia that help to identify GWI phenotypes. Future epidemiological studies will be needed to define the frequency distribution of systemic hyperalgesia in deployed and nondeployed Gulf War era veterans. Prospective studies of veterans from other conflicts may identify subgroups with sudden onset of pain and systemic hyperalgesia, or more gradual progression after toxic or other military exposures. A comprehensive set of Common Data Elements^42^ that assess this wide array of signs, symptoms and organ systems in an interdisciplinary fashion may provide much needed clinical understanding of the overlap between GWI, CFS, FM, sensitization syndromes, migraine, irritable bowel syndrome and affective disorders^1,3,17,34,43–47^ and insights into molecular pathophysiological mechanisms^6^.

Age was not correlated with dolorimetry measurements in any group (Fig. 6). Control women had a wide range of pressure thresholds but did not have any skewed or bimodal distributions that would suggest a trend towards tenderness with increasing age. This is relevant to the development of GWI and CFS/FM. If women who enlisted in the military before 1990 were representative of the general population, then it is reasonable to conclude that some aspect of their 1990-1991 exposures^1^ caused the significant, step-like decrease in dolorimetry thresholds and development of chronic systemic hyperalgesia of GWI. The small modes above 6 kg in GWI and CFS without 1990 FM (Figs. 1 and 5) demonstrate the discontinuity in systemic hyperalgesia that contrasts with the smooth gradient in control subjects. Pathological mechanisms that induce systemic hyperalgesia in GWI women may provide insights into tenderness in CFS/FM and FM. Conversely, one may argue that GWI and CFS women with tenderness originated in the lower left tail of the normal distribution of SC subjects, and that their tenderness at a young age was a risk factor for future development of GWI and CFS. This appears untenable given the high prevalence of GWI in the deployed military and absence of an association of tenderness with age in any group.

There are several limitations to this study. The identical set of questionnaires was not completed by all subjects. This limited the correlations between dolorimetry and these subjective measures, and prevented multivariate analysis. The results cannot be generalized to males. Subjects with inflammatory diseases were excluded based on the CFS criteria, but are commonly included in studies of FM. We did not specifically recruit FM females, and in particular did not recruit using the 2010 or 2011 FM criteria. The outcomes may be modeled to fit the pain, fatigue, sleep and somatic complaints criteria of FM defined by 2010 criteria^6^, but the absence of inquiries into orthostatic intolerance and flu-like complaints precluded assessment of the Canadian Consensus Criteria for ME/CFS^48^ and 2015 SEID^9^ criteria. Separate analyses are needed to assess systemic hyperalgesia in ME/CFS, SEID, and FM defined by 2011 criteria^7^. Common Data Elements^42^ that more fully characterize CFS and GWI symptoms and co-existing disorders such as migraine and irritable bowel syndrome will help to identify relationships with systemic hyperalgesia and disease phenotypes. Other objective outcomes and potential metabolomics biomarkers were not assessed. Future studies will be needed to evaluate potential correlations of dolorimetry with exposure histories, causation, genetic predispositions, lifestyle diatheses, resilience, catastrophizing, childhood abuse, affective and other variables. General linear modeling regression methods will help parse out significant contributors to systemic hyperalgesia versus symptom profiles. Prospective longitudinal studies in large military and civilian populations are needed to evaluate the relationships of these multivariate outcomes to pain and tenderness. This information will generate new hypotheses about neural mechanisms of systemic hyperalgesia.

In conclusion, GWI women have systemic hyperalgesia that correlated with their pain, quality of life, and fatigue ratings (Table 2). Dolorimetry is an inexpensive tool that can be widely taught and deployed as a routine “vital sign” as part of standard care in Department of Veterans Affairs Medical Centers and other clinical systems. This data would provide a database for understanding the development of systemic hyperalgesia in military and civilian populations who are at risk to develop CFS and FM. CFS females had comparable tenderness to GWI, but dolorimetry had lower sensitivity and specificity in CFS. The CFS/FM overlap group have symptoms similar to subjects defined by the 2010 and 2011 FM criteria that include fatigue, cognition, and sleep symptoms, but the distribution of systemic hyperalgesia was not evaluated here using the newer FM criteria. Identification of systemic hyperalgesia as a physical sign is of value because brainstem and descending mechanisms that regulate nociception^49,50^ may be targets for novel therapies to treat pain and tenderness in GWI and CFS.

## Methods

A long term plan was developed to collect pain, fatigue, dolorimetry and other diagnostic features for a large group of GWI, CFS and control subjects, and to analyze these features in cross-sectional fashion. Subjects gave written informed consent to participate in rhinitis, sinusitis, allergy, CFS and GWI studies that were approved by the Georgetown University Institutional Review Board, Department of Defense Congressionally Directed Medical Research Program Human Research Program Office, and registered on clinicaltrials.gov as NCT00810225, NCT00810329, NCT00810368. All clinical investigations were conducted in accordance with the principles expressed in the Declaration of Helsinki.

Participants (n = 1462) were screened using questionnaires^18–21,51,52^, history and physical examinations for diagnosis of CFS^4^, GWI^2,3^ and FM^5^, confirmation of sedentary lifestyle (less than 2 periods of 20 minutes length each for aerobic activity per week), and identification of exclusionary medical or psychiatric conditions^22–26^. FM subjects with autoimmune and other inflammatory diseases were excluded.

CFS was defined using the 1994 Center for Disease Control (CDC) “Fukuda” criteria^4^ of disabling fatigue lasting more than 6 months that cannot be explained by exclusionary medical or psychiatric diagnoses, plus 4 of the 8 ancillary symptoms: myalgia, arthralgia, short term memory or concentration problems, sore throat, sore lymph nodes, headache, sleep disturbance, and post-exertional malaise (exertional exhaustion) (Table 3)^22–24^. Carruthers Canadian Consensus Criteria for Myalgic Encephalomyelitis/Chronic Fatigue Syndrome (ME/CFS) contain the same elements but emphasize post-exertional malaise and add a series of somatosensory and autonomic items that reproduce symptoms of acute flu-like illnesses^48^. In 2015, CFS was reconceptualized by the Institute of Medicine as Systemic Exertion Intolerance Disease (SEID)^9^ (Table 3). Pain was not a component of the SEID definition because there was insufficient evidence in the literature to infer that nociceptive complaints were unique to CFS. Systemic hyperalgesia was evaluated in CFS to clarify this diagnostic issue, and to determine if there was a potential CFS phenotype with systemic pain and tenderness.

Gulf War Illness has developed in 25% to 32% of the 697,000 U.S. military personnel deployed to the Persian Gulf in 1990 to 1991^1^. The rate in nondeployed forces may have been 15%^2^. In 1998 the CDC proposed the Chronic Multisymptom Illness (CMI) criteria that defined cases by having ≥1 chronic symptom from at least 2 of 3 categories (musculoskeletal pain, fatigue, mood-cognition)^2^. The 2000 Kansas criteria were based on symptoms that were significantly more prevalent in deployed than nondeployed personnel^3^. Cases were defined by having symptoms in at least 3 of 6 categories: musculoskeletal pain, fatigue/sleep, neurological/cognitive/mood, respiratory, gastrointestinal, and skin. Symptoms, systemic hyperalgesia^17^, and long term health consequences for female veterans have not been studied as extensively as men^53^.

Fibromyalgia (FM) has been considered the prototypical illness of pain and tenderness^28,54^. Clinical criteria have evolved over the years. The 1990 American College of Rheumatology criteria for FM required widespread pain plus tenderness to thumb pressure at ≥11 of 18 traditional tender points^5^. Pressure should be sufficient to blanch the thumb nail bed, or approximately 4 kg^28^. However, tender point counts may correlate with catastrophizing, general distress, fatigue, depression and sleep alterations, and may be independent of pain^11–15^. The technique is challenging to calibrate and standardize between investigators. Tenderness in FM is present diffusely throughout the body, and is not localized to the 18 specified sites^13^. Therefore, tender point counts were removed from the 2010 revision of the FM criteria^6,55^, even though the concept of tenderness is still considered important for FM diagnosis in clinical practice^6,55–58^. The 2010 revision retained widespread pain, and was expanded to include graded assessments of the severity of fatigue, cognitive difficulties, problems upon waking up, and somatic complaints (Table 1)^14,59^. A modification in 2011 maintained widespread pain, fatigue, cognition, and sleep, but changed somatic complaints to nominal confirmation of headache, lower abdominal pain, and feeling depressed^7^. These modifications increased the overlap between the criteria for FM, CFS and GWI, and blurred distinctions between these clinical entities. We predicted that systemic hyperalgesia, which is typically associated with fibromyalgia, would be more severe in FM than GWI and CFS, and so help resolve differences between these symptom-based case designations.

Dolorimetry was performed with a strain gauge (DPP gauge; Chatillion Products, Ametek Inc, Largo, FL) fitted with a 1 cm^2^ rubber stopper with pressure applied at a rate of 0.5 to 1 kg/s against the 18 traditional tender points^5,12,16^. The end point was the pressure that caused the subject to state that she was experiencing pain. A key aspect was to ensure that the patient felt in control of the process and had trust that the operator would stop pressing as soon as she indicated pain had developed. The mean of the 18 measurements was the dolorimetry pressure threshold. The coefficient of variation for repeated assessments was measured in a subset of women who had serial measurements taken daily by different trained staff members during 3 day in-patient studies.

Preliminary analysis used 2 stratification schemes to classify the 920 qualified women into GWI^2,3^, CFS^4^, FM^5^ and sedentary control (SC) groups. GWI women were deployed to the 1990-1991 Persian Gulf War and met both Chronic Multisymptom Illness (CMI)^2^ and Kansas criteria^3^ (n = 70). Participants who did not meet GWI, CFS or FM criteria were considered to be healthy sedentary control females (SC, n = 428), but included some subjects with chronic idiopathic fatigue and low dolorimetry thresholds without widespread pain. The remaining 422 women met 1994 Fukuda criteria for CFS^4^ and/or 1990 FM criteria of widespread pain plus tenderness at ≥11 of 18 traditional tender points^5^. In Approach 1, subjects were stratified by 1994 CFS criteria to generate CFS_1994_ (n = 366) and FM_1994_ (n = 56) groups. Approach 2 applied 1990 FM criteria^5^ to select CFS_1990_ (n = 170) and FM_1990_ (n = 252) groups. The methods and results were discussed in Supplementary Online Material. The major finding was the large overlap group who met both 1994 CFS and 1990 FM criteria (CFS/FM). Therefore, the final approach defined 5 groups from the combination of the 3 diagnostic criteria: (i) GWI, (ii) CFS only, (iii) FM only, (iv) CFS plus FM (CFS/FM), and (v) SC. Demographics, dolorimety and questionnaire scores were evaluated for each of the 3 approaches.

The primary goal was to determine the frequency distributions of dolorimetry pressure thresholds in order to assess systemic hyperalgesia in GWI, CFS, FM, CFS/FM, and SC groups. Because the women were tested in separate studies, this was a cross-sectional study in groups of convenience and not an epidemiological or longitudinal study.

The secondary goal was to assess the CFS Severity questionnaire^21^, McGill Pain Inventory^18^, Multidimensional Fatigue Inventory (MDFI)^21^, and Medical Outcome Survey Short Form 36 questions (SF-36)^20^ to characterize symptom profiles and disability in each group. Unfortunately, different combinations of questionnaires were used in some studies and some subjects did not complete their forms. As a result, the data were analyzed for univariate correlations and not by multivariate regression.

The third goal was to correlate dolorimetry thresholds with the questionnaire domain scores.

Data were analyzed in SPSS v.22. Group results were compared by ANOVA followed by Tukey Honest Significant Difference and False Discovery Rate to correct for multiple comparisons (p < 0.05), and were reported as mean ± standard deviation. Dolorimetry data were assessed by Kolmogorov-Smirnov tests and were not normally distributed. Nonparametric Kruskal-Wallis tests were used to determine if groups had significant differences. Differences between pairs of groups were determined by Mann-Whitney tests (p < 0.05). Dolorimetry thresholds that distinguished illness groups from sedentary controls were determined by receiver operating characteristics. Dolorimetry was correlated with questionnaire and other variables by Pearson’s method, and explained variances (R^2^) were calculated.

## Supplementary information

Supplementary Information.

Supplementary Dataset 1.

## Data Availability

Dolorimetry, tender point counts, age, CFS Severity Questionnaire, Multidimensional Fatigue Inventory, SF-36 and McGill Total Pain data are appended in the Supplementary Online Material as an Excel file.

## References

[1] Research advisory committee on gulf war veterans’ illnesses. Gulf war illness and the health of gulf war veterans scientific findings and recommendations, https://www.va.gov/RAC-GWVI/docs/Committee_Documents/GWIandHealthofGWVeterans_RAC-GWVIReport_2008.pdf (Accessed 29 Jan 2017) (2008).

[2] Fukuda K (1998). Chronic multisymptom illness affecting air force veterans of the Gulf War. J. A. M. A.

[3] Steele L (2000). Prevalence and patterns of gulf war illness in Kansas veterans: association of symptoms with characteristics of person, place, and time of military service. Am. J. Epidemiol.

[4] Fukuda K (1994). The chronic fatigue syndrome: a comprehensive approach to its definition and study. International chronic fatigue syndrome study group. Ann. Intern. Med.

[5] Wolfe F (1990). The American College of Rheumatology 1990 Criteria for the classification of fibromyalgia. Report of the multicenter criteria committee. Arthritis rheum..

[6] Wolfe F (2010). The American College of Rheumatology preliminary diagnostic criteria for fibromyalgia and measurement of symptom severity. Arthritis Care Res. (Hoboken).

[7] Wolfe F (2011). Fibromyalgia criteria and severity scales for clinical and epidemiological studies: a modification of the ACR preliminary diagnostic criteria for fibromyalgia. J. Rheumatol..

[8] Wolfe F (2016). Revisions to the 2010/2011 fibromyalgia diagnostic criteria. Semin. Arthritis Rheum..

[9] Committee on the diagnostic criteria for myalgic encephalomyelitis/chronic fatigue syndrome, Board on the Health of Select Populations, Institute of Medicine. Beyond myalgic encephalomyelitis/chronic fatigue syndrome. Redefining an illness. Report guide for clinicans, http://www.nationalacademies.org/hmd/~/media/Files/Report%20Files/2015/MECFS/MECFScliniciansguide.pdf. (Accessed November 29, 2017) (2015). 25695122

[10] Latremoliere A, Woolf CJ (2009). Central sensitization: a generator of pain hypersensitivity by central neural plasticity. J. Pain..

[11] Geisser ME (2007). The association between experimental and clinical pain measures among persons with fibromyalgia and chronic fatigue syndrome. Eur. J. Pain.

[12] Croft P, Schollum J, Silman A (1994). Population study of tender point counts and pain as evidence of fibromyalgia. B. M. J.

[13] Petzke F (2001). Dolorimetry performed at 3 paired tender points highly predicts overall tenderness. J. Rheumatol..

[14] Petzke F (2003). What do tender points measure? Influence of distress on 4 measures of tenderness. J. Rheumatol..

[15] Geisser ME (2003). Perception of noxious and innocuous heat stimulation among healthy women and women with fibromyalgia: association with mood, somatic focus, and catastrophizing. Pain..

[16] Naranch K (2002). A tender sinus does not always mean rhinosinusitis. Otolaryngol. Head Neck Surg..

[17] Rayhan RU, Ravindran MK, Baraniuk JN (2013). Migraine in gulf war illness and chronic fatigue syndrome: prevalence, potential mechanisms, and evaluation. Front. Physiol..

[18] Melzack R (1987). The short-form McGill Pain Questionnaire. Pain..

[19] Smets EM, Garssen B, Bonke B, De Haes JC (1995). The multidimensional fatigue inventory (MFI) psychometric qualities of an instrument to assess fatigue. J. Psychosom. Res..

[20] Ware JE, Sherbourne CD (1992). The MOS 36-item short-form health survey (SF-36). I. Conceptual framework and item selection. Med. Care..

[21] Baraniuk JN (2013). A chronic fatigue syndrome (CFS) severity score based on case designation criteria. Am. J. Transl. Res..

[22] Reeves WC (2003). International chronic fatigue syndrome study group. Identification of ambiguities in the 1994 chronic fatigue syndrome research case definition and recommendations for resolution. B. M. C. Health Serv. Res.

[23] Jones JF (2009). An evaluation of exclusionary medical/psychiatric conditions in the definition of chronic fatigue syndrome. B. M. C. Med.

[24] Nater UM (2009). Psychiatric comorbidity in persons with chronic fatigue syndrome identified from the Georgia population. Psychosom. Med..

[25] Reeves WC (2007). Prevalence of chronic fatigue syndrome in metropolitan, urban, and rural Georgia. Popul. Health Metr..

[26] Reeves WC, Lin JM, Nater UM (2013). Mental illness in metropolitan, urban and rural Georgia populations. B. M. C. Public Health..

[27] Triñanes Y, González-Villar A, Gómez-Perretta C, Carrillo-de-la-Peña MT (2014). Profiles in fibromyalgia: algometry, auditory evoked potentials and clinical characterization of different subtypes. Rheumatol. Int..

[28] Bennett RM (1981). Fibrositis: misnomer for a common rheumatic disorder. West. J. Med..

[29] Harris RE (2006). Comparison of clinical and evoked pain measures in fibromyalgia. J Pain..

[30] Petzke F (2003). Increased pain sensitivity in fibromyalgia: effects of stimulus type and mode of presentation. Pain..

[31] Monaco A (2017). Central sensitization-based classification for temporomandibular disorders: A pathogenetic hypothesis. Pain Res. Manag..

[32] Schrepf A (2018). MAPP research network. Sensory sensitivity and symptom severity represent unique dimensions of chronic pain: a MAPP research network study. Pain..

[33] Green, B.G., Mason, J.R, & Kare, M.R. Chemical senses. Volume 2. Irritation. (Marcel Dekker, 1990).

[34] Adam, G. *Visceral perception. Understanding internal cognition*. (Plenum Press, 1998).

[35] Arendt-Nielsen L (2015). Central sensitization in humans: assessment and pharmacology. Handb. Exp. Pharmacol.

[36] Ravindran MK (2011). Migraine headaches in chronic fatigue syndrome (CFS). B. M. C. Neurology.

[37] Baraniuk JN, Clauw JD, Gaumond E (1998). Rhinitis symptoms in chronic fatigue syndrome. Annals Allergy Asthma Immunol.

[38] Baraniuk JN, Naranch K, Maibach H, Clauw D (2000). Irritant rhinitis in allergic, nonallergic, control and chronic fatigue syndrome populations. J. C. F. S.

[39] Baraniuk JN (2005). Neuropathology in rhinosinusitis. Am. J. Respir. Crit. Care Med..

[40] Ravindran MK (2013). Dyspnea in chronic fatigue syndrome (CFS): Comparison of two prospective cross-sectional studies. Global J. Health Sci..

[41] 10.6084/m9.figshare.6653513.v1.

[42] https://www.commondataelements.ninds.nih.gov/MECFS.aspx#tab=Data_Standards.

[43] Bettini L, Moore K (2016). Central sensitization in functional chronic pain syndromes: Overview and clinical application. Pain. Manag. Nurs..

[44] Gracely RH, Schweinhardt P (2015). Programmed symptoms: disparate effects united by purpose. Curr. Rheumatol. Rev.

[45] Yunus MB (2008). Central sensitivity syndromes: a new paradigm and group nosology for fibromyalgia and overlapping conditions, and the related issue of disease versus illness. Semin. Arthritis Rheum..

[46] Maixner W (2016). Overlapping chronic pain conditions: Implications for diagnosis and classification. J. Pain..

[47] Bouin M, Meunier P, Riberdy-Poitras M, Poitras P (2001). Pain hypersensitivity in patients with functional gastrointestinal disorders: a gastrointestinal-specific defect or a general systemic condition?. Dig. Dis. Sci.

[48] Carruthers BM (2007). Definitions and aetiology of myalgic encephalomyelitis: how the Canadian consensus clinical definition of myalgic encephalomyelitis works. J. Clin. Pathol..

[49] Barnden LR (2018). Hyperintense sensorimotor T1 spin echo MRI is associated with brainstem abnormality in chronic fatigue syndrome. Neuroimage Clin..

[50] Nakatomi Y (2014). Neuroinflammation in patients with chronic fatigue syndrome/myalgic encephalomyelitis: An ¹¹C-(R)-PK11195 PET Study. J. Nucl. Med..

[51] Rayhan RU (2013). Administer and collect medical questionnaires with Google documents: a simple, safe, and free system. Appl. Med. Inform.

[52] Baraniuk JN (2013). Carnosine treatment for gulf war illness: a randomized controlled trial. Glob. J. Health Sci..

[53] Coughlin SS (2017). A review of epidemiologic studies of the health of gulf war women veterans. J. Environ. Health Sci.

[54] Häuser W (2015). Fibromyalgia. Nat. Rev. Dis. Primers.

[55] Vanderschueren S, Van Wambeke P, Morlion B (2010). Fibromyalgia: do not give up the tender point count too easily: comment on the article by Wolfe *et al*. Arthritis Care. Res. (Hoboken)..

[56] Sarzi-Puttini, P. *et al*. Are the ACR 2010 diagnostic criteria for fibromyalgia better than the 1990 criteria? *Autoimmun. Rev*. **17**, 33–35 (2018). 10.1016/j.autrev.2017.11.007 29108831

[57] Able, S. L. *et al*. Variations in the management of fibromyalgia by physician specialty: rheumatology versus primary care. *Pragmat. Obs. Res.* **7**, 11–20 (2016). 10.2147/POR.S79441 PMC5085275 27799842

[58] Martin, S. A., Coon, C. D., McLeod, L. D., Chandran, A. & Arnold, L. M. Evaluation of the fibromyalgia diagnostic screen in clinical practice. *Journal of Evaluation in Clinical Practice* **20**(2), 158–165 (2014). 10.1111/jep.12102 24283211

[59] Smith, H. S., Harris, R. & Clauw, D. Fibromyalgia: an afferent processing disorder leading to a complex pain generalized syndrome.* Pain Physician*. **14**, E217–45 (2011). 21412381

