# Supplementary material for: Systemic Hyperalgesia in Females with Gulf War Illness, Chronic Fatigue Syndrome and Fibromyalgia
Source: Sci Rep. 2020 Apr 1;10:5751. doi: 10.1038/s41598-020-62771-9 (PMC7113257; doi:10.1038/s41598-020-62771-9)
Supplement: Supplementary file 1 — Supplementary Information. [file 41598_2020_62771_MOESM1_ESM.pdf]

Supplementary Online Material

Systemic Hyperalgesia in Females with Gulf War Illness, Chronic Fatigue Syndrome and Fibromyalgia

Amber A. Surian
James N. Baraniuk *

* Corresponding Author
Email baraniuj@georgetown.edu
Division of Rheumatology, Immunology and Allergy
Georgetown University
3800 Reservoir Road NW
Washington DC 20007-2197
TEL 1-202-687-8231
FAX 1-202-687-9886

INTRODUCTION

There is confusion about the designation of CFS and FM because by the overlap of fatigue, cognition, sleep, widespread pain, and somatic complaints in the 1994 CDC "Fukuda" criteria for CFS, [1] and 2010 and 2011 American College of Rheumatology criteria for FM. [2-4] The Carruthers Canadian Consensus Criteria [5] expanded the list of symptomatic complaints and emphasized post-exertional malaise as a key diagnostic feature of CFS. The 2015 Institute of Medicine report introduced Systemic Exertion Intolerance Disease (SEID) [6] based on fatigue, post-exertional malaise, sleep, cognition and orthostatic intolerance dysfunction, but removed pain as a component because pain did not distinguish CFS from fibromyalgia.

The role of systemic hyperalgesia, or tenderness, in these illnesses was assessed using dolorimetry. Two stratification algorithms were used to subdivide a large group of female participants in ongoing clinical research studies. The pressures that caused pain were then compared between subgroups.

Approach 1 identified women with GWI and sedentary controls (SC), then selected the CFS group using the 1994 CFS criteria (CFS_{1994} group) [4]. The FM_{1994} women had pain and tenderness but did not meet 1994 CFS criteria that included post-exertional malaise, cognitive and sleep dysfunction. This approach did not rely on tenderness as a selection criteria for CFS.

In contrast, Approach 2 used the 1990 FM criteria [5] of widespread pain and tenderness to thumb pressure to select the FM_{1990} group who had substantial systemic hyperalgesia. Women who did not meet the 1990 FM criteria but did satisfy the 1994 CFS criteria were the CFS_{1990} group. The same SC group was used.

Approaches 1 and 2 show the differences in symptom profiles that result when using CFS-biased and FM-biased selection criteria.

METHODS

Approach 1

Approach 1 stratified women based on the 1994 Fukuda CFS criteria [1] without regard to tenderness. This is relevant to clinical research and practice when all females with CFS are clustered into a single group. For clarity, groups were labelled by subscript 1994 ($_{1994}$).

(i) GWI women were identified by history of deployment to the 1990-1991 Persian Gulf War and meeting Chronic Multisymptom Illness (CMI) [7] and Kansas criteria. [8]

(ii) The 1994 Fukuda criteria [1] positively selected women with CFS (CFS_{1994} group). All 70 GWI females also met CFS criteria.

(iii) Subjects who did not meet CFS or GWI criteria were assessed for 1990 FM criteria of widespread pain plus tenderness at ≥ 11 of 18 traditional tender points [9] to identify females with fibromyalgia characteristics (FM_{1994} group). FM_{1994} women were not typical of other fibromyalgia studies because everyone meeting CFS criteria, including those with widespread pain and tenderness, were selected as CFS_{1994} and so were excluded from the FM_{1994} group (Table S1).

(iv) The remaining women were considered to be healthy sedentary control females (SC). Some SC subjects had chronic idiopathic fatigue or low dolorimetry thresholds without widespread pain.

The primary goal of Approach 1 was to determine the frequency distributions of dolorimetry pressure thresholds in GWI, CFS_{1994}, FM_{1994} and SC groups. Because the women were tested once in separate studies, this was a cross-sectional study in patient groups of convenience.

The secondary goal was to assess the CFS Severity questionnaire, [10] McGill Pain Inventory, [11] Multidimensional Fatigue Inventory (MDFI), [12] and Medical Outcome Survey Short Form 36 questions (SF-36) [13] to characterize symptom profiles and disability in each group. Unfortunately, different combinations of questionnaires were used in some studies and some subjects did not complete their forms. As a result, these data were not analyzed by multivariate regression.

The third goal was to correlate dolorimetry thresholds with each of the questionnaire domain scores.

Approach 2

Approach 2 relied on the 1990 FM criteria. [9] Groups were designated by subscript 1990 ($_{1990}$). This approach relied on counting the number of FM tender points and so had an intrinsic bias to include women with systemic hyperalgesia in the FM_{1990} group. This approach did not rely on fatigue, cognitive or sleep criteria that are part of CFS [1] and FM 2010 and 2011 criteria. [2,3]

(i) GWI women [7,8] were again separated.

(ii) The 1990 FM criteria of widespread pain and tenderness to thumb pressure at ≥ 11 of 18 traditional tender points [9] identified the FM_{1990} female group.

The remaining subjects were assessed by 1994 Fukuda CFS criteria [1] to select:

(iii) CFS_{1990} and,

(iv) sedentary control women (SC) who were the same as Approach 1.

Frequency distributions for dolorimetry and questionnaire domain scores were assessed as in Approach 1.

Statistics

Data were analyzed in SPSS v.22. Group results were compared by ANOVA followed by Tukey Honest Significant Difference and False Discovery Rate, and reported as mean ± standard deviation. As shown in the main approach (Table 1), dolorimetry was not normally distributed and so Kruskal-Wallis and Mann-Whitney tests were used for nonparametric comparisons. Dolorimetry thresholds that distinguished illness groups from sedentary controls were determined by receiver operating characteristics. Dolorimetry was correlated with questionnaire and other variables by Pearson's method, and explained variances (R^2) calculated.

RESULTS

Approach 1: 1994 CFS criteria

Average age was in the 5th decade, but GWI women were older than SC (Table S2). CFS_{1994} had the highest proportion of Caucasians.

Table S1. Approach 1 algorithm and demographics.

Entry criterion	Females with dolorimetry measurements			
Exclusions	Chronic medical or psychiatric diseases			
GWI status	Gulf War exposures in 1990 & 1991 + Kansas GWI criteria			
	Yes	No		
	GWI	Not GWI		
CFS status Fukuda Criteria, 1994		6 months of disabling fatigue without explanation plus ≥ 4 of 8 ancillary criteria		
	Yes	Yes	No	
	GWI	CFS_{1994}	Not GWI or CFS	
1990 FM status	Not assessed a priori		Widespread pain + Tender points by thumb pressure	
			<11/18	≥11/18
Groups	**GWI**	**CFS_{1994}**	**SC**	**FM_{1994}**
N	70	366	428	56
Age (years)	48.2 ± 11.4 *	45.3 ± 11.6	42.7 ± 13.5	43.3 ± 14.8
% Caucasian	65.2%	81.0%	60.2%	69.5%
Dolorimetry (kg)	2.9 ± 1.6 *†‡	4.3 ± 2.2 *	7.2 ± 2.4	3.9 ± 1.4 *
Mann Whitney vs SC	p<0.0001	p<0.0001	Kruskal-Wallis vs. SC k=4 p<0.0001	p<0.0001
Mann Whitney vs CFS_{1994}	p<0.0001	Kruskal-Wallis vs. CFS_{1994} k=3 p<0.0001	excluded	p=0.14
Mann Whitney vs FM_{1994}	p<0.0001			
Range	0.2 to 7.2	0.5 to 12.3	0.4 to 12.5	0.6 to 9.8
Median	2.6	3.9	6.9	3.6
1st quartile	1.8	2.9	5.5	2.9
3rd quartile	3.5	5.6	8.8	4.4
Skewness	0.936	0.865	0.125	1.542
Kurtosis	0.555	0.714	-0.580	5.658
Tender point counts (0-18)	12.7 ± 5.1 *†	9.9 ± 5.9 *	3.3 ± 3.9	11.4 ± 4.6 *
McGill Pain Total Score	21.5 ± 11.9 *†‡ n=57	14.6 ± 9.1 * n=90	3.2 ± 6.3 ‡ n=68	9.4 ± 7.6 *† n=25

First, symptoms were assessed to identify CFS_{1994} and GWI women. Second, the remaining subjects were divided into FM_{1994} and SC based on 1990 FM criteria. Third, dolorimetry thresholds and other variables were assessed between groups (mean ± SD). Significantly different by ANOVA and Tukey HSD<0.05 compared to: *SC, †CFS_{1994}, ‡FM_{1994}.

Bins of 0.5 kg were used to rank subjects for frequency analysis. Distributions for dolorimetry results were not normal by one-sample Kolmogorov-Smirnov tests with Lilliefois corrections (p<0.028) but were skewed to the right (Table S1). SC females had a very wide and squat frequency distribution (7.2 ± 2.4 kg, mean±SD, Table S1) that extended from 0.4 to 12.5 kg (Figure S1). Pain thresholds were significantly lower

in GWI, CFS$_{1994}$ and FM$_{1994}$ than SC by Kruskal-Wallis and Mann-Whitney tests ($p<0.0001$) (Table S1). GWI (2.9 ± 1.6 kg) was significantly lower than CFS$_{1994}$ (4.3 ± 2.2 kg), while FM$_{1994}$ had an intermediate level (3.7 ± 1.2 kg) that was not different from GWI or CFS$_{1994}$. Receiver operating characteristics defined dolorimetry thresholds of ≤ 4.5 kg for GWI (sensitivity = 0.857, specificity = 0.853) and FM$_{1994}$ (sensitivity = 0.786, specificity = 0.853), and ≤ 5.5 kg for CFS$_{1994}$ (sensitivity = 0.730, specificity = 0.743) (Figure S1, Table S2).

Figure S1. Dolorimetry frequency analysis using bins of 0.5 kg in women stratified according to 1994 CFS criteria.

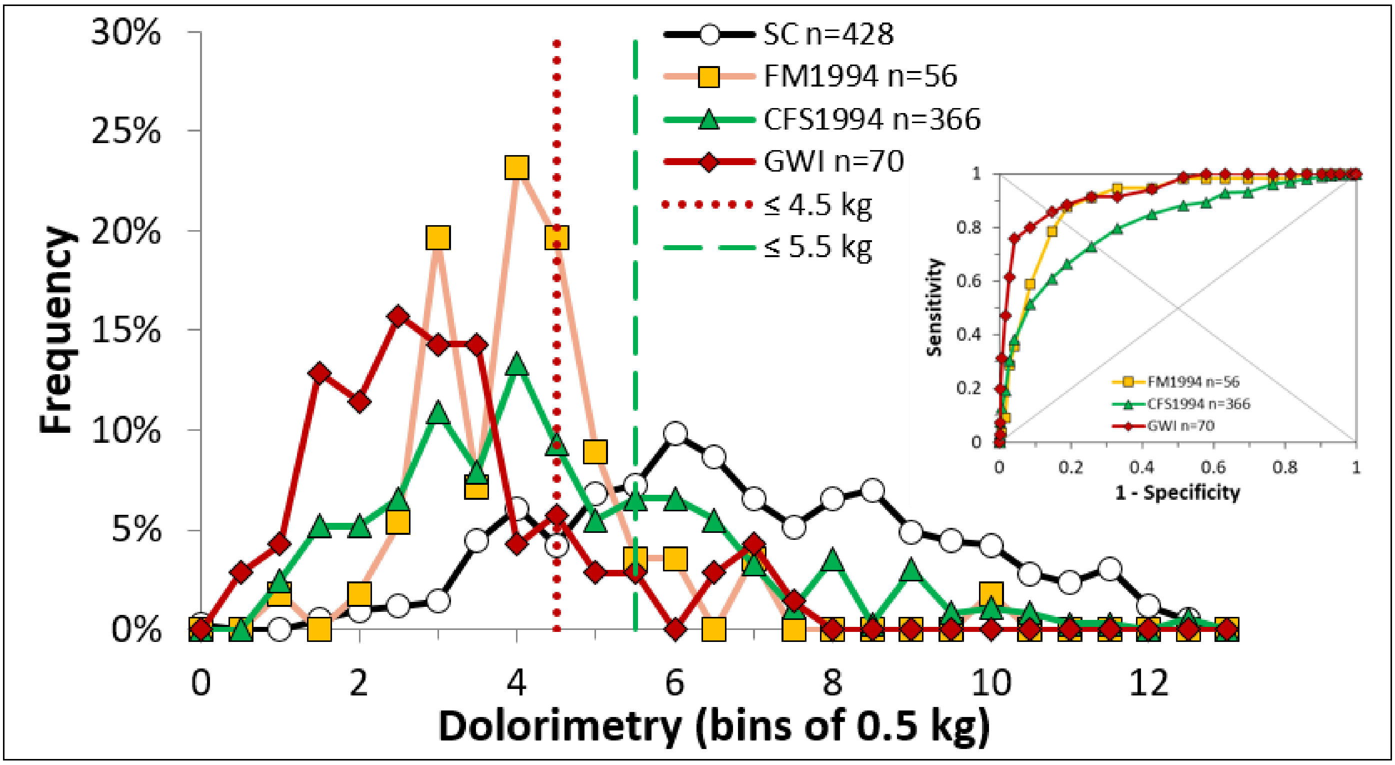

The distributions of average pressure thresholds causing pain were shifted to the left in GWI (red diamonds and line), CFS$_{1994}$ (green triangles and line) and FM$_{1994}$ (orange squares and line) compared to SC females (white circles, black line). Thresholds of 4.5 kg for GWI and FM$_{1994}$ (vertical red dotted line), and 5.5 kg for CFS$_{1994}$ (vertical green dashed line) were defined by receiver operating characteristics (inset).

Table S2. Receiver operating characteristics from Figure S1. Thresholds for each group were in bold.

ROC	GWI n=70	CFS$_{1994}$ n=366	FM$_{1994}$ n=56	SC = 428; Specificity
4	0.800	0.516	0.589	0.914
4.5	**0.857**	0.609	**0.786**	**0.853**
5	0.886	0.664	0.875	0.811
5.5	0.914	**0.730**	0.911	**0.743**
AUC	0.951	0.851	0.928	
Concordance	0.853	0.737	0.845	

GWI and CFS$_{1994}$ had equivalent scores for Chronic Fatigue Symptom Questionnaire items that were significantly higher than both FM$_{1994}$ and SC (Figure S2). FM$_{1994}$ had higher severities than SC for Fatigue, Myalgia and Arthralgia.

Figure S2. Chronic Fatigue Symptom Questionnaire.

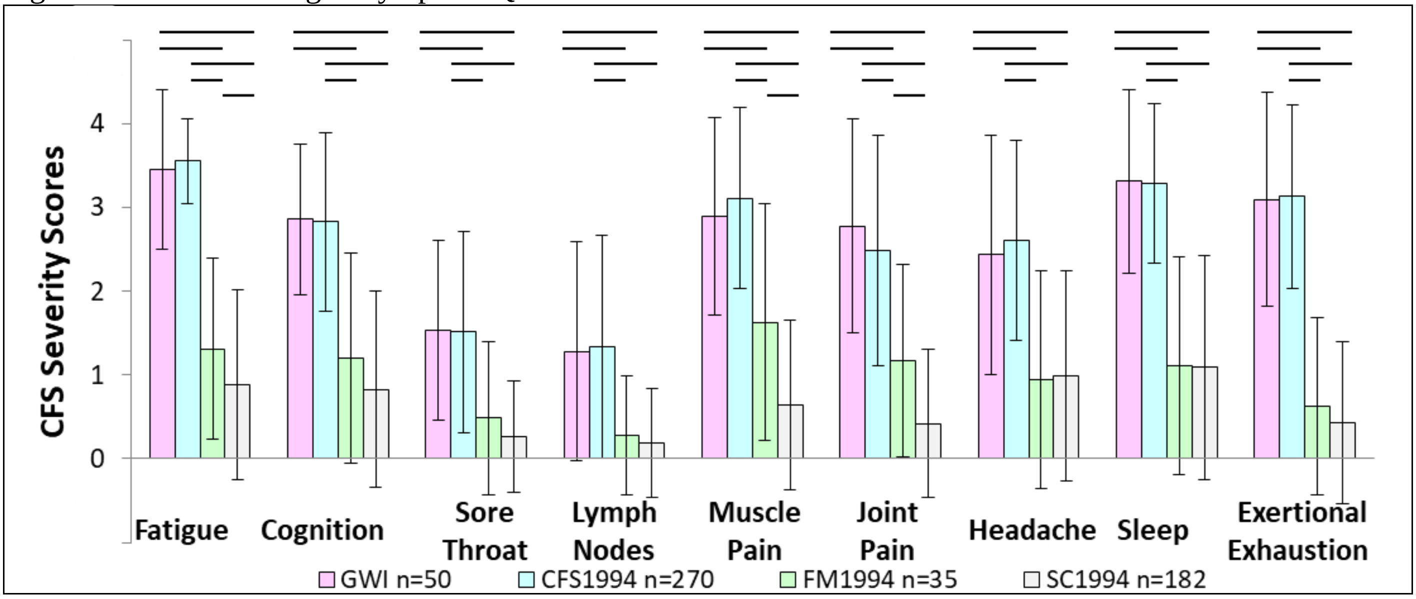

Symptom severity over the past 6 months was compared for Fatigue and the 8 ancillary symptoms (mean ± SD). Lines above the bars indicate significant differences between groups by ANOVA ($p<0.05$) followed by Tukey's Honest Significant Difference (<0.05) plus FDR (<0.005) to correct for all data comparisons.

Multidimensional Fatigue Inventory domain scores were equivalent for GWI and CFS_{1994}, and significantly higher than SC and FM_{1994} (Figure S3). FM_{1994} had higher scores than SC for General Fatigue, Physical Fatigue and Mental Fatigue.

Figure S3. Multidimensional Fatigue Inventory Domain scores (mean ± SD).

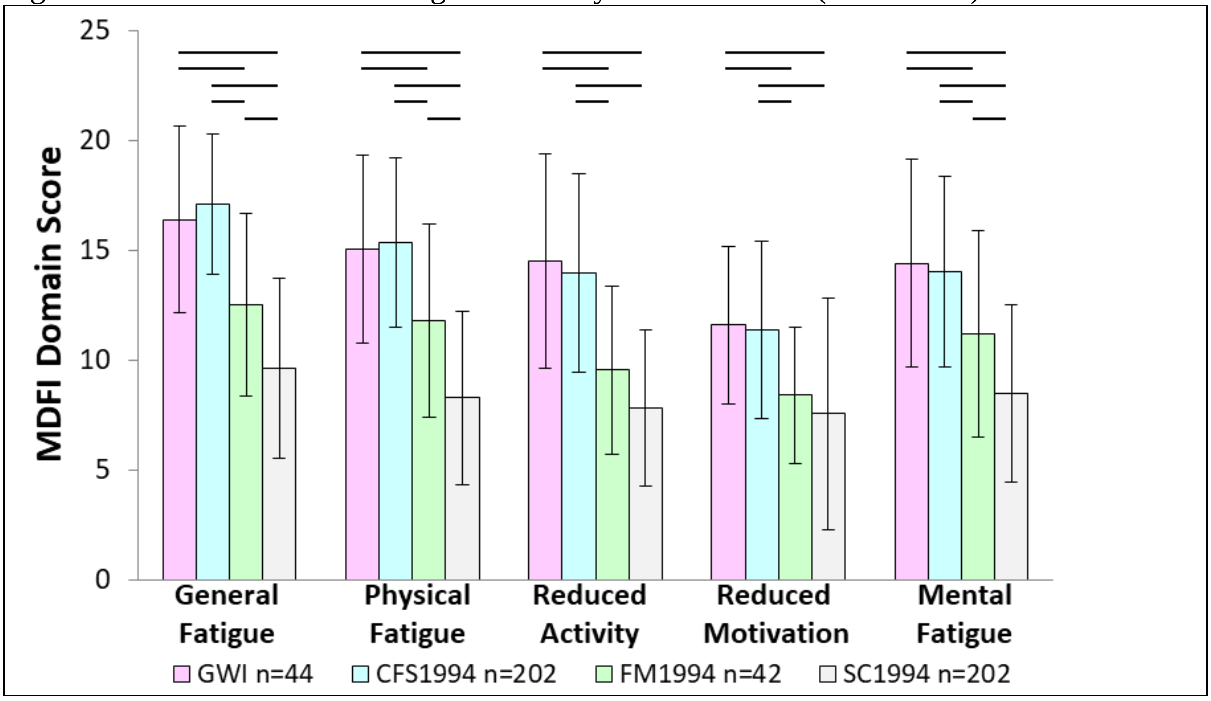

Lines above the bars indicate significant differences between groups by ANOVA followed by Tukey's Honest Significant Difference (<0.05) plus FDR (<0.001) to correct for all data comparisons.

SC had the highest SF-36 domain scores indicating better quality of life (Figure S4). GWI and CFS_{1994} had equivalent low scores for Physical Function, Social Function, Role-Physical, Vitality and General Health. Bodily Pain was significantly lower in GWI, CFS_{1994} and FM_{1994} compared to SC women.

Unlike the other quality of life domains, scores for Role-Emotional in GWI, and Mental Health in CFS_{1994} were significantly lower than SC.

Figure S4. SF-36 Domain scores (mean ± SD).

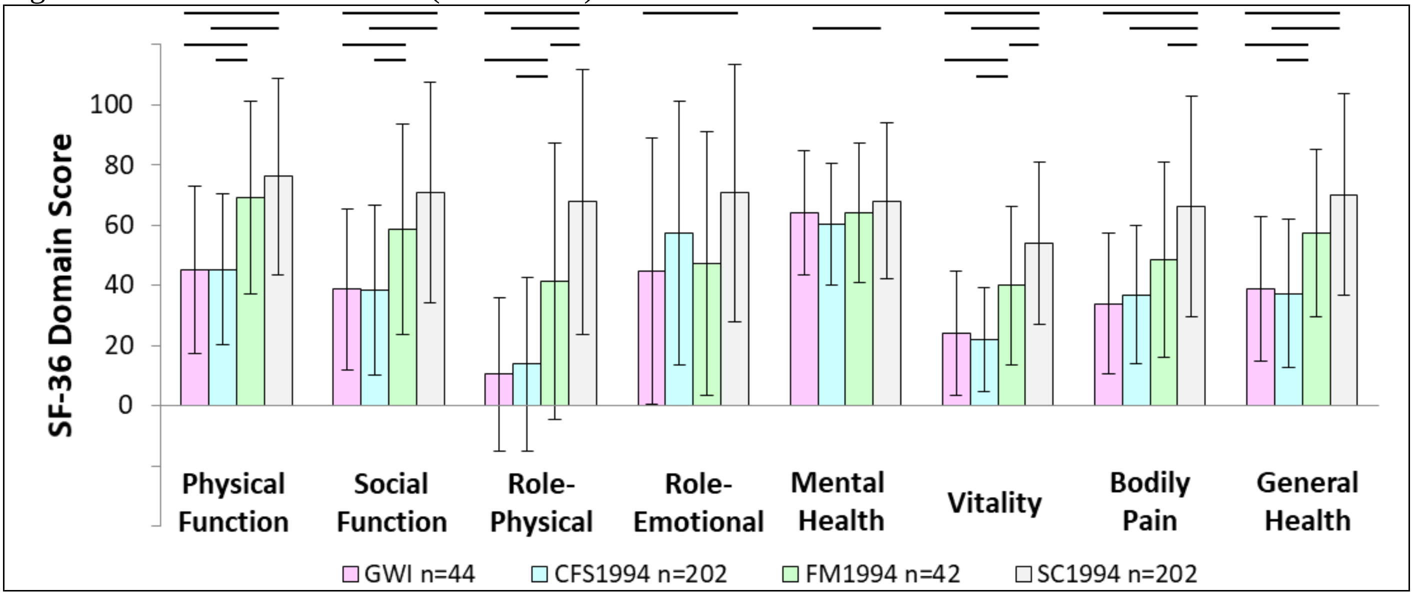

Lines above the bars indicate significant differences between groups by ANOVA followed by Tukey's Honest Significant Difference (<0.05) plus FDR (<0.005) to correct for all data comparisons.

Pain thresholds (kg) measured by dolorimetry were highly correlated with the number of tender points determined by thumb pressure ($R^2 \geq 0.479$) (Table S3). GWI women had explained variances between 0.25 and 0.5 for measures of pain (McGill Total Pain Score, SF-36 Bodily Pain, CFS questionnaire Joint Pain, and Muscle Pain), and fatigue (CFS questionnaire Exertional Exhaustion and Fatigue, and MDFI Physical Fatigue). In contrast, explained variances for CFS_{1994}, FM_{1994} and SC were < 0.2 for all of these variables. Important negative findings were the absence of correlations between pain thresholds and age, sleep, cognition, and Mental Health. Because different combinations of questionnaires were used in each of the studies that contributed data to this analysis, it was not possible to model pain thresholds and other variables by multivariate regression or other methods.

Table S3. Explained variances (R^2) from Pearson correlations between dolorimetry (kg) and domain scores for each group in Approach 1.

Variable	GWI	CFS_{1994}	FM_{1994}	SC
Tender point count	0.479	0.691	0.507	0.567
≥11/18 tender points	0.338	0.356	0.189	0.179
Widespread pain	0.372	0.158	0.079	0.000
McGill Total Pain	0.320	0.172	0.096	0.083
SF36				
Physical Functioning	0.354	0.121	0.001	0.000
Bodily Pain	0.328	0.077	0.000	0.004
Social Functioning	0.271	0.041	0.011	0.001
Role-Physical	0.245	0.045	0.022	0.002
Role-Emotional	0.164	0.001	0.006	0.004
General Health	0.121	0.019	0.002	0.002
Mental Health	0.098	0.034	0.000	0.001
Vitality	0.063	0.048	0.003	0.002
CFS Symptom Severity Scores				
Joint pain	0.457	0.029	0.001	0.035
Muscle pain	0.437	0.139	0.162	0.070
Exertional exhaustion	0.377	0.025	0.167	0.007
Fatigue	0.256	0.012	0.011	0.029
Disturbed sleep	0.162	0.005	0.045	0.011
Throat	0.145	0.003	0.002	0.036
Sore lymph nodes	0.101	0.032	0.011	0.014
Headache	0.081	0.013	0.064	0.001
Memory & concentration	0.020	0.025	0.003	0.000
Multidimensional Fatigue Inventory				
Reduced Activity	0.302	0.017	0.000	0.009
Physical Fatigue	0.269	0.004	0.009	0.017
Reduced Motivation	0.188	0.008	0.000	0.015
General Fatigue	0.180	0.014	0.003	0.000
Mental Fatigue	0.050	0.000	0.012	0.000
Age	0.006	0.001	0.001	0.004

Age did not correlate with dolorimetry in any of these cross-sectional groups (horizontal regression lines in Figure S5). Dolorimetry thresholds were highest in SC_{1994} women over the entire age range. There was insufficient information to assess potential changes in pressure thresholds as a function of the duration of GWI, CFS_{1994}, or FM_{1994} symptoms, or years since medical diagnosis.

Figure S5. Age distributions of dolorimetry pain thresholds for each group in Approach 1.

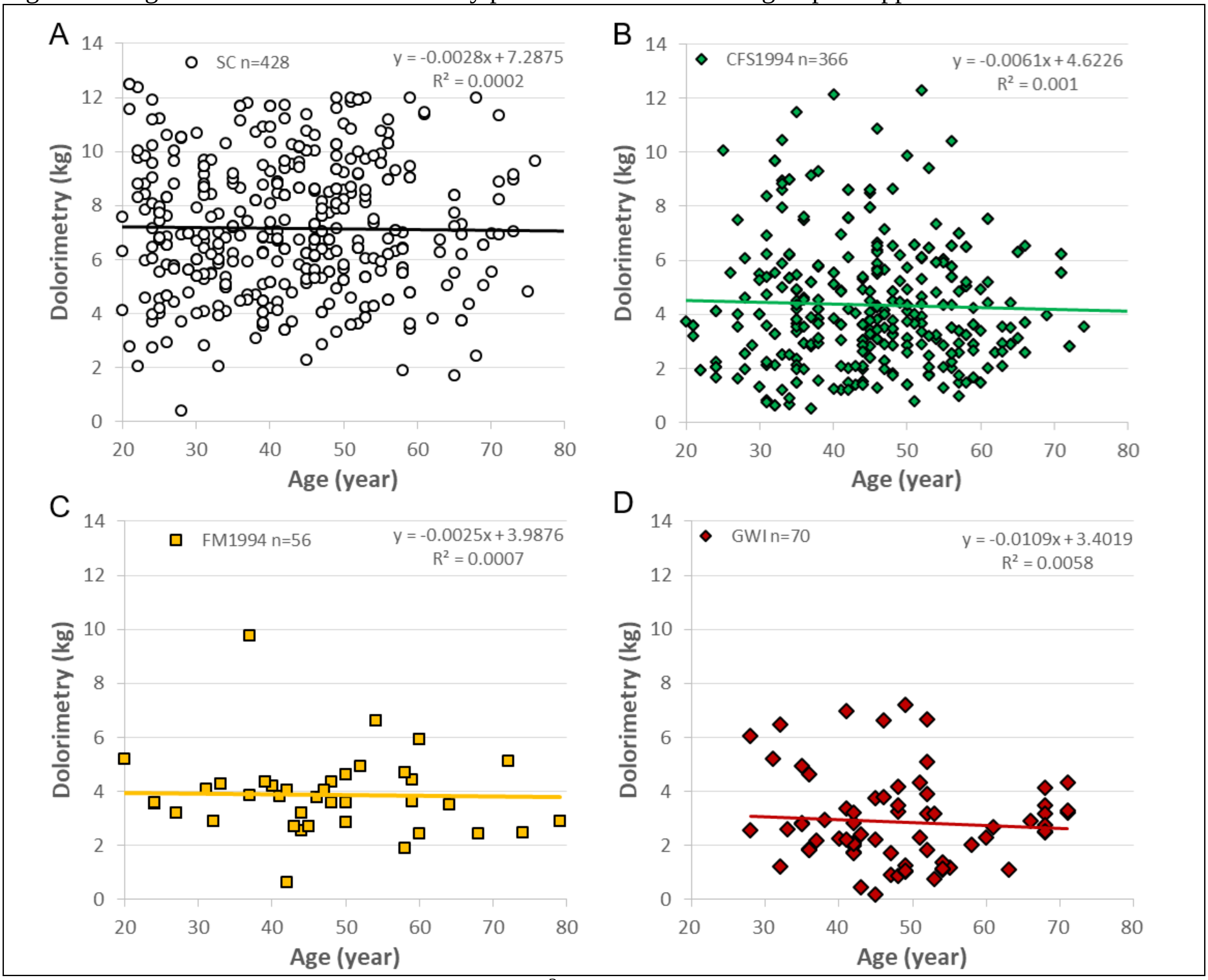

All linear regression lines were horizontal with $R^2<0.01$ (not significant) indicating no correlations for dolorimetry with age. Sedentary Control (SC, open circles and black line, A) had the highest thresholds. Data were shifted downwards (e.g. y-intercepts) for CFS_{1994} (B, green), FM_{1994} (C. orange) and GWI (D, red). Equations for regression lines and explained variances were provided in the upper right corner of each graph.

Approach 2: 1990 FM criteria

Primary stratification by the 1990 FM criteria of widespread pain and tenderness to thumb pressure [9] shifted 196 women from the CFS_{1994} into the FM_{1990} group (Tables S1 and S4). McGill Pain Total Score, a proxy for widespread pain, was ranked GWI > FM_{1990} = CFS_{1990} > SC. Tender point counts were ranked GWI = FM_{1990} > CFS_{1990} > SC. The transfer of CFS_{1994} women into the FM_{1990} group increased tender point counts from 12.1 ± 3.5 in FM_{1994} to 13.4 ± 4.5 in the FM_{1990} group, and increased McGill Total Pain scores from 8.9 ± 7.6 to 14.0 ± 8.6, respectively. Tender point counts decreased from 9.9 ± 5.9 in CFS_{1994} to 5.7 ± 2.1 in CFS_{1990}, but McGill Total Pain scores were equivalent (14.6 ± 9.1 and 13.1 ± 10.2, respectively). This transfer of women who met both CFS and FM criteria indicated the importance of the CFS/FM overlap group, and need to better understand of the interactions between mechanisms responsible for systemic hyperalgesia, pain, fatigue, cognition, sleep dysfunction.

Table S4. Approach 2: 1990 FM criteria.

Entry criterion	Females with dolorimetry measurements			
Exclusions	Chronic medical or psychiatric diseases			
GWI Status	Gulf War exposures in 1990 & 1991 + Kansas GWI criteria			
	GWI	Not GWI		
1990 FM status		Widespread pain + Tender points by thumb pressure		
		<11/18		≥11/18
		Not GWI, Not FM_{1990}		FM_{1990}
CFS status Fukuda Criteria, 1994		6 months of disabling fatigue without explanation plus 4 of 8 ancillary criteria		
		Yes	No	
Groups	**GWI**	**CFS_{1990}**	**SC**	**FM_{1990}**
N	70	170	428	252
Age (years)	48.2 ± 11.4 *	45.0 ± 10.9	42.7 ± 13.5	45.7 ± 12.5
% Caucasian	65.2%	85.6%	60.2%	75.0%
Dolorimetry (kg)	2.9 ± 1.6 *†‡	5.8 ± 2.1*	7.2 ± 2.4	3.2 ± 1.4 *†
Mann Whitney vs SC	p<0.0001	p<0.0001	Kruskal-Wallis vs. SC k=4 p<0.0001	p<0.0001
Mann Whitney vs CFS_{1990}	p<0.0001	Kruskal-Wallis vs. CFS_{1990} k=3 p<0.0001	excluded	p<0.0001
Mann Whitney vs FM_{1990}	p=0.014			
Range	0.2 to 7.2	1.4 to 12.3	0.4 to 12.5	0.5 to 9.8
Median	2.6	5.5	6.9	3.1
1st quartile	1.8	4.4	5.5	2.3
3rd quartile	3.5	6.6	8.8	3.8
Skewness	0.936	0.748	0.125	0.950
Kurtosis	0.555	0.583	-0.580	2.106
Tender point counts (0-18)	12.7 ± 5.1 *†	5.0 ± 3.7 *	3.3 ± 3.9	12.9 ± 4.7 *†
McGill Pain Total Score	21.5 ± 11.9 *†‡ n=57	12.2 ± 8.7 * n=26	3.2 ± 6.3 n=68	14.2 ± 9.2 * n=82

First, GWI females were selected. Second, the 1990 FM criteria of widespread pain and tenderness [2] were used to identify the FM_{1990} group. Third, the remaining subjects were assessed by Fukuda CFS criteria [1] to designate the CFS_{1990} group. The residual women were sedentary healthy controls (SC). Dolorimetry thresholds and other variables were assessed between groups (mean ± SD). Significantly different by ANOVA and Tukey HSD<0.05 compared to: * SC, † CFS_{1990}, ‡ FM_{1990}.

Dolorimetry pressures causing pain were ranked SC > CFS_{1990} > FM_{1990} > GWI ($p<0.014$ by Mann-Whitney tests). None of the frequency distribution curves were normal by one-sample Kolmogorov-Smirnov tests with Lilliefois corrections ($p<0.028$). The FM_{1994} (Figure S1) and FM_{1990} (Figure S6) frequency distributions were highly skewed and narrow (high kurtosis) suggesting that these fibromyalgia subjects were a distinct subgroup with systemic hyperalgesia and were not the tail of the normal population distribution that merged gradually with the bulk of the distribution.

The dolorimetry thresholds for GWI and FM_{1990} remained at 4.5 kg based on receiver operating characteristics (Table S5). The CFS_{1990} group had a rightward shift towards the sedentary control group compared to CFS_{1994}, and an increase in dolorimetry threshold to 6 kg by receiver operating characteristics (Figure S6).

The CFS_{1990} group was comparable to the group who met only CFS but not FM criteria in the main analysis ("CFS").

By design, the GWI and SC groups were the same in Approaches 1 and 2.

Figure S6. Dolorimetry frequency analysis using bins of 0.5 kg and Approach 2 based on 1990 FM criteria.

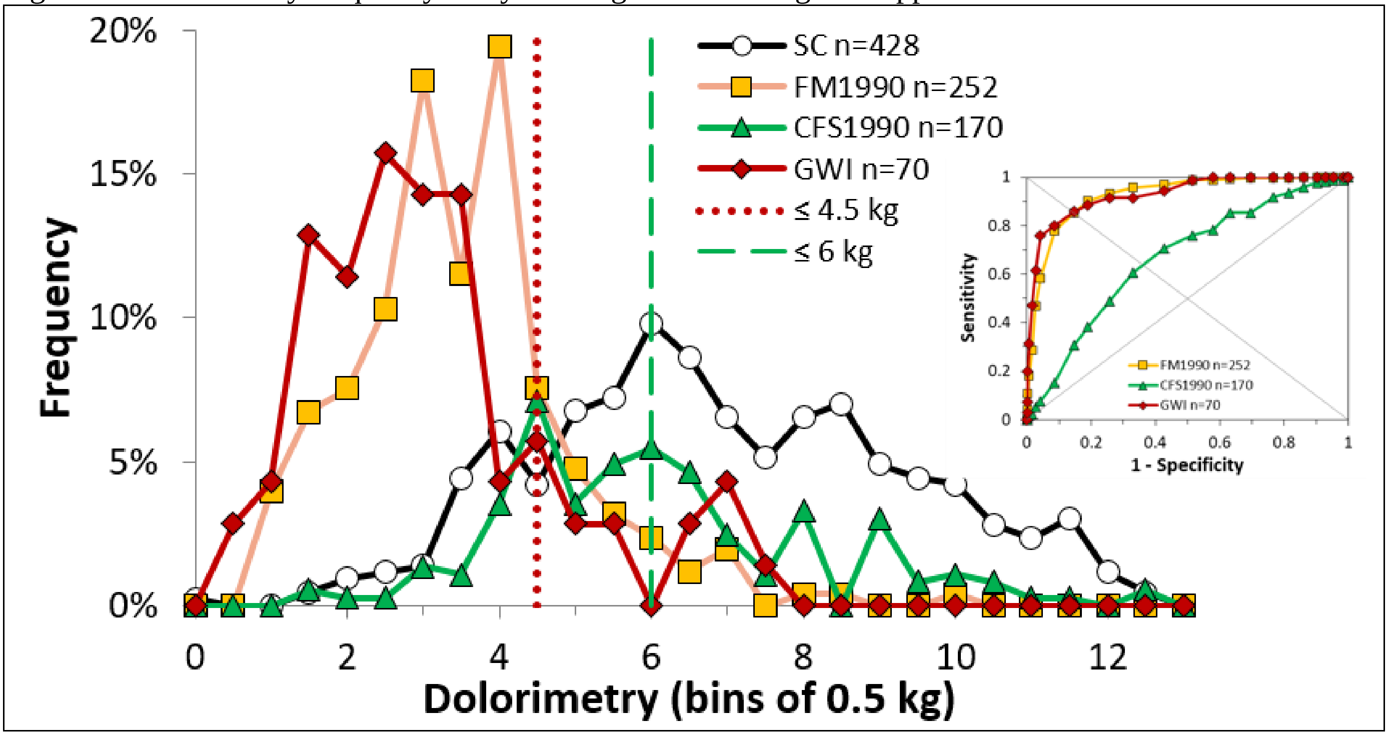

The distributions of average pressure thresholds causing pain were shifted to the left in GWI (red diamonds and line) and FM_{1990} (yellow squares and line) compared to SC (white circles, black line). CFS_{1990} (green triangles and line) were shifted towards healthy females. Thresholds defined by receiver operating characteristics (insert) were 4.5 kg for GWI and FM_{1990} (vertical red dotted line), and 6 kg for CFS_{1990} (vertical green dashed line).

Table S5. Receiver operator characteristics for groups defined by 1990 FM criteria in Figure S6. Significant thresholds were in bold.

ROC	GWI n=70	CFS_{1990} n=170	FM_{1990} n=252	SC = Specificity
4.5	**0.857**	0.306	**0.853**	**0.853**
5	0.886	0.382	0.901	0.811
5.5	0.914	0.488	0.933	0.743
6	0.914	**0.606**	0.956	**0.671**
AUC	0.951	0.727	0.952	
Concordance	0.853	0.641	0.853	

Symptom scores

Chronic Fatigue Symptom Questionnaire scores were equivalent in GWI, CFS_{1990} and FM_{1990} except for minor differences from CFS_{1990} for muscle and joint pain. These scores were all significantly higher than SC (Figure S7).

Figure S7. Chronic Fatigue Symptom Questionnaire for Approach 2.

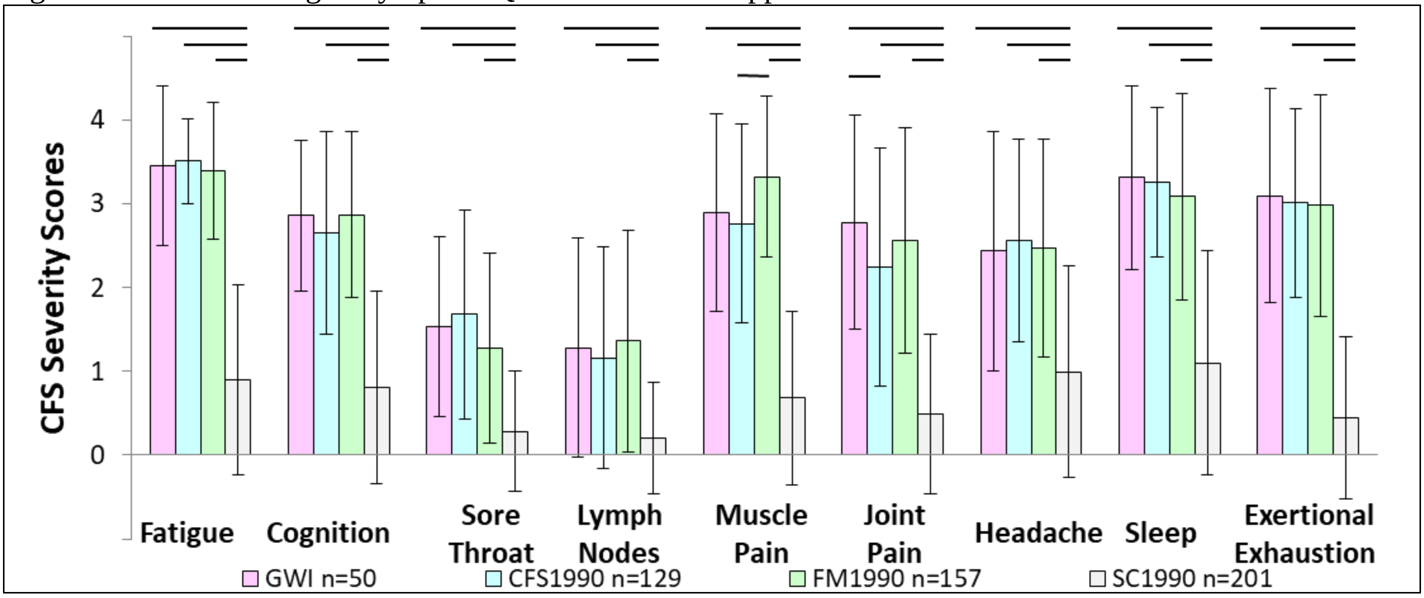

Symptom severity over the past 6 months was compared for fatigue and the 8 symptoms (mean ± SD) based on 1990 FM criteria. Lines above the bars indicate significant differences between groups by ANOVA ($p<0.05$) followed by Tukey's Honest Significant Difference (<0.05) plus FDR (<0.02) to correct for all data comparisons.

Multidimensional Fatigue Inventory domain scores were equivalent for GWI, CFS_{1990}, and FM_{1990}, and significantly higher than SC (Figure S8).

Figure S8. Multidimensional Fatigue Inventory Domain scores (mean ± SD) for Approach 2.

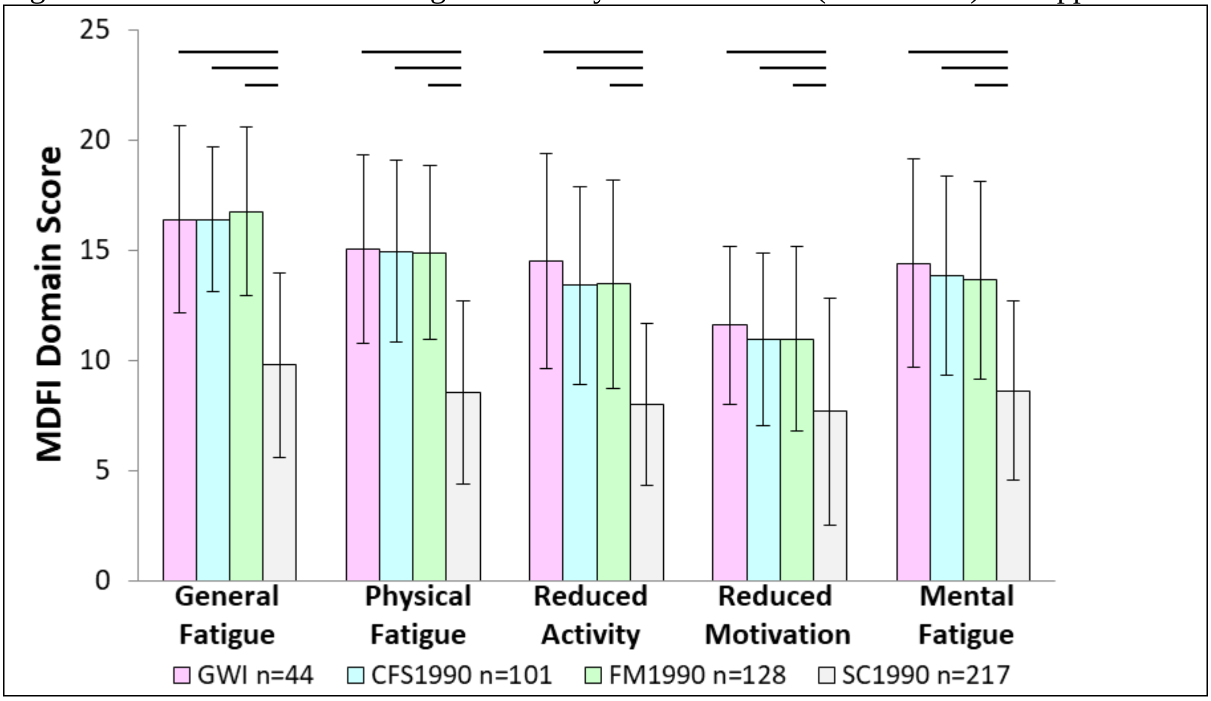

Lines above the bars indicate significant differences between groups by ANOVA followed by Tukey's Honest Significant Difference (<0.05) plus FDR ($<10^{-6}$) to correct for all data comparisons.

SF-36 domain scores were highest in SC indicating better quality of life (Figure S9). The exceptions were Mental Health which was not different between groups, and Role Emotional where SC was higher than GWI. Scores were equivalent between GWI, CFS_{1990} and FM_{1990} indicating that Approach 2 could not distinguish between groups by quality of life.

Figure S9. SF-36 Domain scores (mean ± SD) for Approach 2.

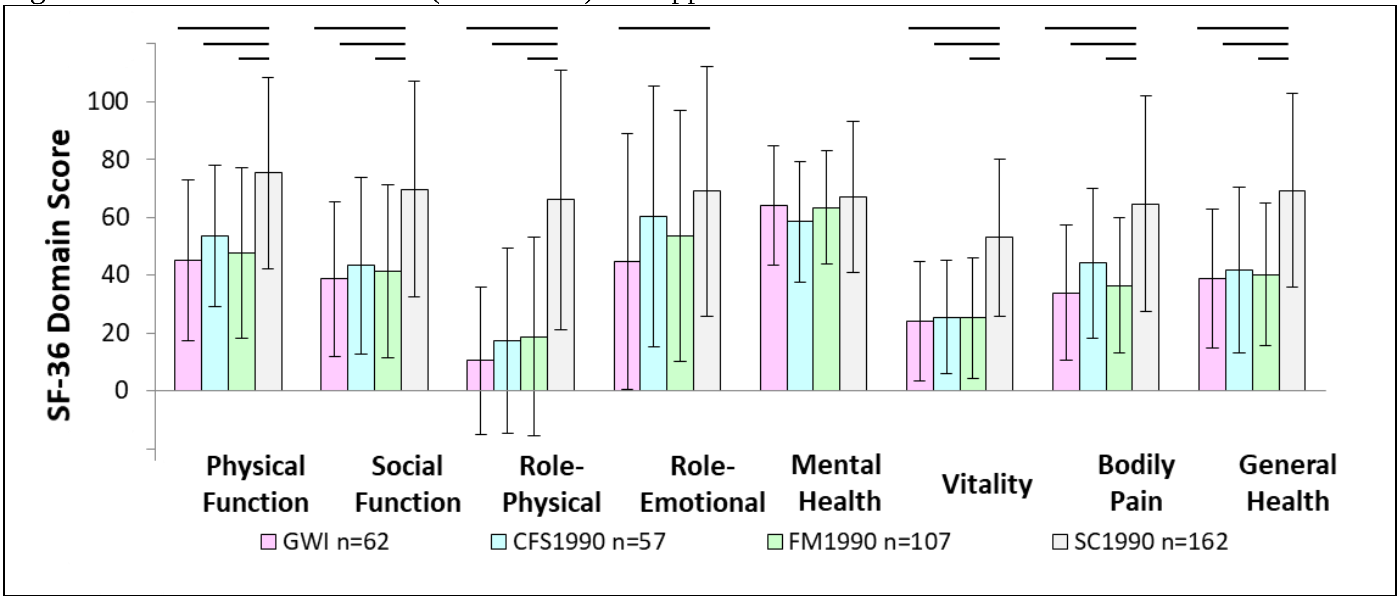

Lines above the bars indicate significant differences between groups by ANOVA followed by Tukey's Honest Significant Difference (<0.05) plus FDR (<0.01) to correct for all data comparisons.

Dolorimetry pain thresholds (kg) were correlated with the number of tender points determined by thumb pressure in all 4 groups (Table S6). However, only GWI had meaningful correlations ($R^2 > 0.2$) with pain, physical functioning and other variables.

Table S6. Explained variances (R^2) from Pearson correlations between dolorimetry (kg) and domain scores for each group stratified by 1990 FM criteria in Approach 2.

Variable	GWI	CFS_{1990}	FM_{1990}	SC
Tender point count	0.479	0.602	0.509	0.645
≥11/18 tender points	0.372	0.048	0.154	0.001
Widespread pain	0.338	0.076	0.178	0.243
McGill Total Pain	0.320	0.164	0.171	0.112
SF36				
Physical Functioning	0.354	0.026	0.114	0.000
Bodily Pain	0.328	0.013	0.063	0.010
Social Functioning	0.271	0.005	0.097	0.003
Role-Physical	0.245	0.046	0.061	0.007
Role-Emotional	0.164	0.011	0.001	0.009
General Health	0.121	0.008	0.102	0.003
Mental Health	0.098	0.075	0.002	0.004
Vitality	0.063	0.008	0.050	0.006
CFS Symptom Severity Scores				
Joint pain	0.457	0.001	0.044	0.050
Muscle pain	0.437	0.035	0.144	0.070

Exertional exhaustion	0.377	0.005	0.052	0.008
Fatigue	0.256	0.000	0.031	0.024
Disturbed sleep	0.162	0.002	0.056	0.009
Throat	0.145	0.000	0.006	0.033
Sore lymph nodes	0.101	0.017	0.032	0.012
Headache	0.081	0.033	0.008	0.001
Memory & concentration	0.020	0.000	0.053	0.001
Multidimensional Fatigue Inventory				
Reduced Activity	0.302	0.001	0.027	0.017
Physical Fatigue	0.269	0.012	0.058	0.037
Reduced Motivation	0.188	0.001	0.013	0.019
General Fatigue	0.180	0.010	0.027	0.001
Mental Fatigue	0.050	0.031	0.031	0.002
Age	0.006	0.007	0.008	0.000

Main Approach

Receiver Operating Characteristics for Main Approach (Table S7). GWI and CFS/FM had the lowest threshold for dolorimetry and systemic hyperalgesia. FM defined by 1990 criteria [5] but without CFS [3] had a threshold of 4.5 kg. CFS without 1990 FM had a threshold of 6 kg that was significantly differentiated CFS from SC.

Table S7. Receiver operating characteristics for Figure 1. Thresholds for each group were in bold.

Threshold	Sensitivity				Specificity
Kg	GWI	CFS/FM	FM	CFS	SC
≤4	**0.800**	**0.832**	0.589	0.153	0.914
≤4.5	0.857	0.872	**0.786**	0.306	0.853
≤6	0.914	0.959	0.946	**0.606**	0.671
AUC	0.905	0.906	0.848	0.607	

Only the GWI group had correlations between dolorimetry and symptoms with R2 > 0.25 as shown by the relationship between the sum of myalgia and arthralgia scores from the CFS Symptom Severity questionnaire in the main approach data (Fig S10).

Figure S10. Pearson correlations between the sum of myalgia and arthralgia scores and dolorimetry.

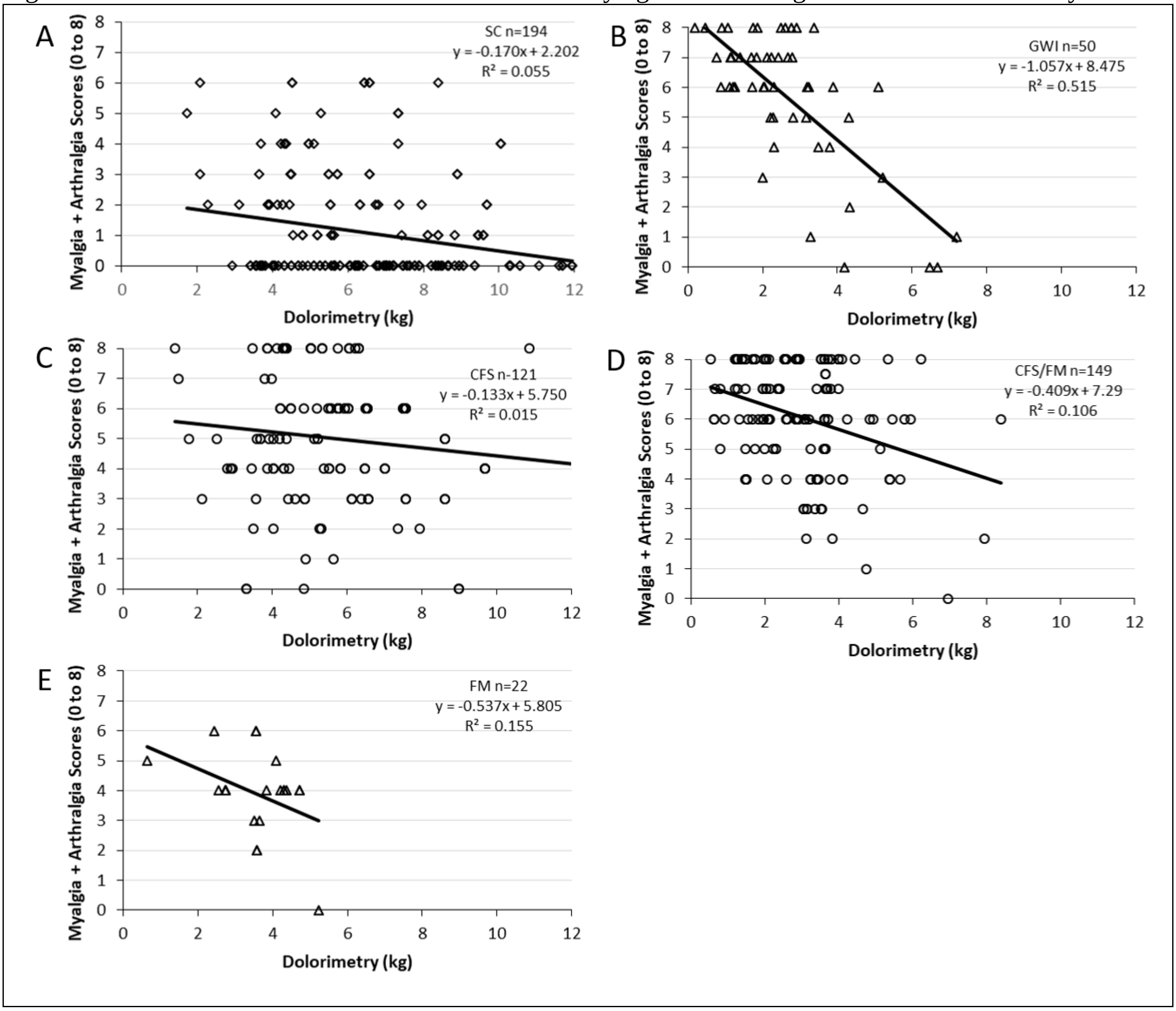

DISCUSSION

In Approach 1, the 1994 CDC criteria [1] and 2000 Kansas criteria [8] were used to select CFS_{1994} and GWI groups before dolorimetry was assessed. GWI had significantly lower dolorimetry pressures, higher tender point counts and McGill Total Pain scores than CFS_{1994}. Other subjective scores were not significantly different (Figures S2 to S4). The diagnosis of CFS and GWI did not require tenderness, but both groups had significant systemic hyperalgesia (Figure S1). The GWI group was the most tender, and had a ROC defined threshold of 4.5 kg (Figure S1). Dolorimetry pressures were correlated with the number of traditional thumb pressure points (Table S3) where the pressure required to blanch the thumb nail bed was about 4 kg. However, the cutoff of ≥11/18 tender points was less effective (Table S3). Receiver operating characteristics indicated this physical sign of systemic hyperalgesia had approximately 85% sensitivity and specificity to confirm the diagnosis of GWI in Gulf War era female veterans who had systemic complaints (Table S2).

CFS_{1994} had equivalent tenderness to GWI women (Table S1), but dolorimetry had lower sensitivity and specificity (Table S2). Despite the similarity of symptom scores, CFS and GWI women had different patterns of correlations between dolorimetry and symptoms (Table S3) suggesting they were distinctly different disease entities. The tenderness suggests that the 2015 Institute of Medicine criteria for Systemic Exertion Intolerance Disease (SEID) [6] may need to be modified to consider systemic hyperalgesia as a pathological mechanism in CFS.

Approach 1 purposefully selected the FM subgroup by (i) exclusion from the CFS group because they did not meet the 1994 Fukuda criteria [1], and (ii) inclusion using the 1990 American College of Rheumatology criteria [9] of both widespread pain and ≥ 11 of 18 thumb pressure tender points. Because the FM_{1994} women had CFS questionnaire item scores that were intermediate between SC_{1994} (floor) and GWI and CFS_{1994} (ceiling) (Figure S7), they were unlikely to have sufficient fatigue, cognitive, sleep, visceral or somatic pain symptoms to meet the 2010 or 2011 FM criteria. [2,3]

Approach 2 selected women based on 1990 FM criteria of widespread pain and tenderness to thumb pressure. [9] Dolorimetry and tender point counts were comparable for GWI and FM_{1990}, with CFS_{1990} having intermediate levels. McGill Total Pain was worse in GWI than the other 3 groups (Table S4). Selection by tender point counts failed to distinguish between GWI, CFS_{1990} and FM_{1990} for CFS symptoms (Figure S7), MDFI domain scores (Figure S8) and SF-36 quality of life scores (Figure S9).

In conclusion, Approach 1 used the 1994 CFS criteria to stratify groups based on fatigue and other symptoms. Dolorimetry and McGill Total Pain Scores were significantly worst for GWI, and equivalent between FM_{1994} and CFS_{1994} compared to the normal SC_{1994} group. However, GWI and CFS_{1994} had significantly worse results for the other subjective symptom scores. FM_{1994} scores were intermediate between these and SC_{1994}.

Approach 2 selected group based on tenderness (1990 FM criteria). GWI had the highest McGill pain scores. GWI and FM_{1990} had equivalent low dolorimetry measurements, with intermediate levels for CFS_{1990}. However, GWI, CFS_{1990}, and FM_{1990} were equivalent for other pain, fatigue, and quality of life variables.

The large group of women meeting both 1994 CFS and 1990 FM criteria accounted for the differences between the approaches, and were investigated using the main approach.

DATA AVAILABILITY

Dolorimetry, tender point counts, age, CFS Severity Questionnaire, Multidimensional Fatigue Inventory, SF-36 and McGill Total Pain data are appended in the Supplementary Online Material as an Excel file.

REFERENCES

1. Fukuda, K., *et al*. The chronic fatigue syndrome: a comprehensive approach to its definition and study. International Chronic Fatigue Syndrome Study Group. *Ann Intern Med*. **121**, 953-959 (1994).

2. Wolfe, F., *et al*. The American College of Rheumatology preliminary diagnostic criteria for fibromyalgia and measurement of symptom severity. *Arthritis Care Res. (Hoboken)*. **62**, 600-610 (2010).

3. Wolfe, F., *et al*. Fibromyalgia criteria and severity scales for clinical and epidemiological studies: a modification of the ACR Preliminary Diagnostic Criteria for Fibromyalgia. *J. Rheumatol*. **38**, 1113-1122 (2011); doi: 10.3899/jrheum.100594.

4. Smith, H. S., Harris, R., & Clauw D. Fibromyalgia: an afferent processing disorder leading to a complex pain generalized syndrome. *Pain Physician*. **14**, E217-45 (2011).

5. Carruthers BM. Definitions and aetiology of myalgic encephalomyelitis: how the Canadian consensus clinical definition of myalgic encephalomyelitis works. *J. Clin. Pathol. 60*, 117-119 (2007).

6. Committee on the Diagnostic Criteria for Myalgic Encephalomyelitis/Chronic Fatigue Syndrome, Board on the Health of Select Populations, Institute of Medicine. Beyond Myalgic Encephalomyelitis/Chronic Fatigue Syndrome. Redefining an Illness. Report Guide for Clinicans. 2015. http://www.nationalacademies.org/hmd/~/media/Files/Report%20Files/2015/MECFS/MECFScliniciansguide.pdf. Accessed November 29, 2017

7. Fukuda, K., *et al*. Chronic multisymptom illness affecting Air Force veterans of the Gulf War. *J. A. M. A.* **280**, 981-988 (1998).

8. Steele L. Prevalence and patterns of Gulf War illness in Kansas veterans: association of symptoms with characteristics of person, place, and time of military service. *Am. J. Epidemiol*. **152**, 992-1002 (2000).

9. Wolfe, F., *et al*. The American College of Rheumatology 1990 Criteria for the Classification of Fibromyalgia. Report of the Multicenter Criteria Committee. *Arthritis Rheum*. **33**, 160-172 (1990).

10. Baraniuk, J. N., *et al*. A Chronic Fatigue Syndrome (CFS) severity score based on case designation criteria. *Am. J. Transl. Res*. **5**, 53-68 (2013).

11. Melzack, R. The short-form McGill Pain Questionnaire. *Pain*. **30**, 191-197 (1987).

12. Smets, E. M., Garssen, B., Bonke, B., & De Haes, J. C. The Multidimensional Fatigue Inventory (MFI) psychometric qualities of an instrument to assess fatigue. *J. Psychosom. Res*. **39**, 315-325 (1995).

13. Ware, J. E., Jr., & Sherbourne, C. D. The MOS 36-item short-form health survey (SF-36). I. Conceptual framework and item selection. *Med. Care*. **30**, 473-483 (1992)
